# Strong Anionic Fluorene Donor–Acceptor Copolyelectrolytes from Protected Hydrophobic Precursors

**DOI:** 10.1002/marc.202400925

**Published:** 2025-01-02

**Authors:** Anton H. Hofman, Peter Dijkstra, Marleen Kamperman

**Affiliations:** ^1^ Polymer Science, Zernike Institute for Advanced Materials University of Groningen Nijenborgh 3 Groningen 9747 AG The Netherlands

**Keywords:** conjugated polyelectrolytes, conducting materials, polyfluorenes, protecting groups, Suzuki polycondensation

## Abstract

Conjugated polyelectrolytes (CPEs), materials that are defined by a π‐conjugated backbone and charged ionic functional groups, are frequently prepared through direct polymerization of charged monomer species in aqueous media. This route is, however, often accompanied by labor‐intensive work‐up procedures, low yields, and ultimately results in materials that are difficult to characterize. To overcome these inconveniences, in this work protection chemistry is applied on sulfonate‐functionalized fluorene monomers that are polymerized under standard Suzuki polycondensation conditions to obtain protected donor–acceptor copolymers. Treatment of the organo‐soluble precursors under nucleophilic conditions resulted in quantitative removal of the protecting groups, thereby exposing the strong anionic functionalities. Unlike in other studies, the conjugated backbones remained unharmed during this process: the photophysical properties of the CPEs are identical to their hydrophobic precursors.

## Graphical abstract

Anionic fluorene‐containing donor–acceptor copolyelectrolytes are synthesized using a protection/deprotection strategy. After copolymerization of the protected fluorene monomers under standard Suzuki polycondensation conditions, the protecting groups can be quantitatively removed from the hydrophobic precursor. While the solubility of the material changed drastically after exposing the negatively charged groups, the photophysical properties remained unchanged.

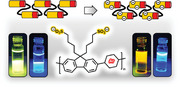

## 1 Introduction

Conjugated polyelectrolytes (CPEs) are polymeric materials that combine the properties of conjugated polymers, polymers that are defined by an electronically delocalized π‐conjugated backbone, and the high charge density of polyelectrolytes.^[^ ^1^, ^2^ ^]^ The addition of ionic groups renders these semiconducting materials soluble in polar organic solvents, and in certain cases even water. CPEs have found application in various optoelectronic devices, including OLEDs^[^ ^3^, ^4^ ^]^ and OPVs.^[^ ^5^ ^]^ Besides their use in electronics, the high charge density also allows one to prepare π‐conjugated ionic complexes by addition of an oppositely charged species. Examples include CPE‐surfactant complexes,^[^ ^6^ ^]^ aqueous light‐harvesting systems from donor‐acceptor couples,^[^ ^7^ ^]^ and concentrated semiconducting fluids through polyelectrolyte coacervation.^[^ ^8^, ^9^ ^]^ Other innovative applications of CPEs have been reported as well, ranging from photocatalysts for hydrogen production,^[^ ^10^ ^]^ chemical sensors,^[^ ^11^ ^]^ and photosensitizers for generating antimicrobial hydrogels.^[^ ^12^ ^]^

Over the years, most attention has been given to CPEs that are based on a conventional π‐conjugated backbone functionalized with pendant ionic groups.^[^ ^13^, ^14^ ^]^ Within this family, there seems to be a strong preference for permanently (i.e., pH‐independent) charged groups: sulfonates in case of anionic CPEs and quaternary ammonium salts in case of cationic CPEs.^[^ ^15^ ^]^

The strong polycations are typically synthesized via post‐modification of either amine‐ or bromo‐functionalized polymers through quaternization reactions using alkyl halides^[^ ^3^, ^16^ ^]^ or amines,^[^ ^10^, ^17^ ^]^ respectively. Strong polyanions have proven themselves more challenging, since mild and efficient post‐polymerization reactions with the aim of introducing sulfonate groups have not yet been reported. Therefore, the majority of the strong anionic CPEs are directly synthesized from charged monomers.^[^ ^5^, ^9^, ^18^, ^19^ ^]^ However, due to their poor solubility and charged nature, molecular weights are frequently limited, characterization is complicated, work‐up can be a tedious process, and the procedure is often accompanied by low yields. Additionally, purification sometimes even leads to undesired and uncontrolled doping.^[^ ^20^ ^]^

Application of protection chemistry on sulfonates may be an interesting alternative, since removal of relatively labile groups from a protected polymeric precursor is more effective than introducing new groups via polymer‐analogues reactions that are difficult to drive to completion.^[^ ^21^, ^22^ ^]^ Indeed, some protection chemistry has recently been used to produce sulfonate‐functionalized CPEs, although in these studies polymerization of the protected monomers was sometimes problematic, proof for quantitative removal of the protecting groups was not given, and the photophysical properties of the charge‐neutral precursor and the CPE were not directly compared or were not the same.^[^ ^23^, ^24^, ^25^, ^26^, ^27^ ^]^ An overview that allows straightforward comparison of this work to previous literature where protection/deprotection chemistry was applied is given in the Supporting Information (Table S4). This table lists the type of CPE, the protecting group, the deprotection route and efficiency, and whether deprotection affected the photophysical properties or not.

In view of the need of a more robust and less destructive route, we here report the successful translation of our previously developed protection/deprotection strategy for the synthesis of well‐defined poly(3‐sulfopropyl methacrylates) to conjugated polymers,^[^ ^28^, ^29^ ^]^ using polyfluorenes as model system.^[^ ^30^ ^]^ The protected sulfonate‐functionalized F4SN monomers (“fluorene‐butyl‐sulfonic ester‐neopentyl”) were copolymerized with aromatic (Ar) comonomers via Suzuki polycondensation to produce several PF4SN‐Ar donor–acceptor precursors, and deprotection was performed under nucleophilic conditions which resulted in quantitative cleavage of the sulfonic esters groups, thereby exposing the charged moieties. Most importantly, the CPE's photophysical properties remained unaffected throughout this process.

## 2 Results and Discussion

**Scheme 1:**
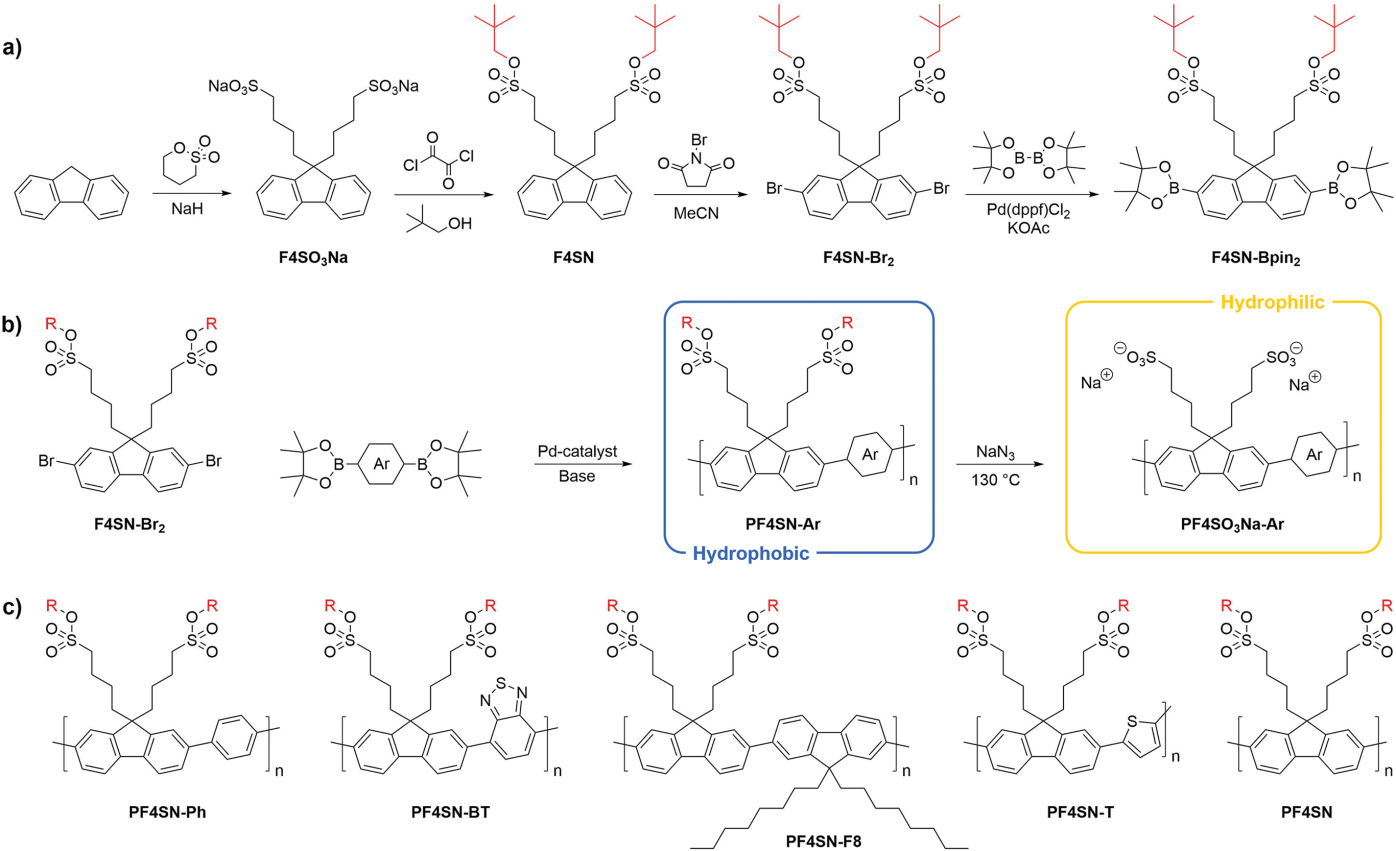
Reaction scheme describing the synthesis of a) protected monomers F4SN‐Br_2_ and F4SN‐Bpin_2_, and b) PF4SO_3_Na‐Ar anionic CPEs and their protected charge‐neutral hydrophobic PF4SN‐Ar precursors prepared via Suzuki polycondensation. c) Chemical structures of the five PF4SN copolymers described in this work. R = neopentyl.

The protected dibromo‐functionalized fluorene monomer, F4SN‐Br_2_ was synthesized via a three‐step route as described in **Scheme** **1**a. First, both sulfonate groups were installed via a ring‐opening reaction of 1,4‐butane sultone using sodium hydride as base. Then the neopentyl protecting groups were introduced in a two‐step, one‐pot reaction; this particular group was selected because of its high thermal stability and its inertness towards bases, acids, and weak nucleophiles,^[^ ^31^ ^]^ conditions that are frequently encountered during the preparation of conjugated polymers. Other protecting groups, such as isobutyl and phenyl esters, are unsuitable due to their lower thermal and chemical stability.^[^ ^32^ ^]^ Double protection of the fluorene monomer could only be achieved when the precursor F4SO_3_Na was base‐ and moisture‐free. Finally, both bromo groups were added to F4SN using *N*‐bromosuccinimide (60 °C, acetonitrile as solvent). Experimental details and characterization of all intermediates are provided in the Supporting Information (Figures S1– S3).

Next, polymerization of the protected monomer F4SN‐Br_2_ was performed under standard Suzuki polycondensation conditions, using an aqueous base, a Pd catalyst, and a diboronic acid bis(pinacol) ester comonomer (Scheme 1b).^[^ ^33^, ^34^, ^35^ ^]^ For initial investigations, 1,4‐benzenediboronic acid bis(pinacol) ester (Ph‐Bpin_2_) was selected: it is commercially available, the polymer properties are well understood,^[^ ^36^ ^]^ and it is sufficiently small to not have significant impact on the solubility properties compared to large alkyl‐substituted comonomers. Various settings were tested with the aim to maximize both the yield and molecular weight. The best results were obtained at 90 °C, 24 h, using Pd(PPh_3_)_4_ as catalyst, 4.0 M aqueous K_2_CO_3_ with the addition of a phase‐transfer catalyst (A336), and a 1,4‐dioxane/*o*‐xylene (1/1) solvent mixture (entry 4 in Table S1, Supporting Information). Such a mixture was necessary to dissolve the rather polar polymer and to simultaneously avoid water from entering the organic phase; either pure 1,4‐dioxane and *o*‐xylene are not suitable. After optimization PF4SN‐Ph was obtained with good yields (> 80 %) and reasonable molecular weights (Figure S5, Supporting Information). Most importantly, the neopentyl protecting groups remained intact throughout the polymerization reaction. The characteristics of the PF4SN‐Ph copolymer that was used for further investigation are listed in **Table** **1**.

**Table 1:** Overview of the synthesized PF4SN copolymers. Yields were determined gravimetrically, molecular weights $M_{n}$ (kg mol^−1^) and their distribution (Đ) by GPC (chloroform, PS standards), glass transition temperatures ($T_{g}$) by DSC, and the absorption $\lambda_{\max}$ (abs) and emission maxima $\lambda_{\max}$ (em) by UV–Vis and PL spectroscopy (chloroform solutions), respectively.

Entry	Polymer	Yield [%]	$M_{n}$	Đ	$T_{g}$ [°C]	$\lambda_{\max}$ [abs] [nm]	$\lambda_{\max}$ [em] [nm]
1	PF4SN‐Ph	83	7.64	2.28	122	368	409
2	PF4SN‐BT	82	7.83	2.21	114	320, 443	535
3	PF4SN‐F8	87	11.61	2.18	90	386	416, 440
4	PF4SN‐T	75	6.30	2.28	n.d.	429	473, 495
5	PF4SN	74	9.60	2.03	101	380	415, 438

**Figure 1:**
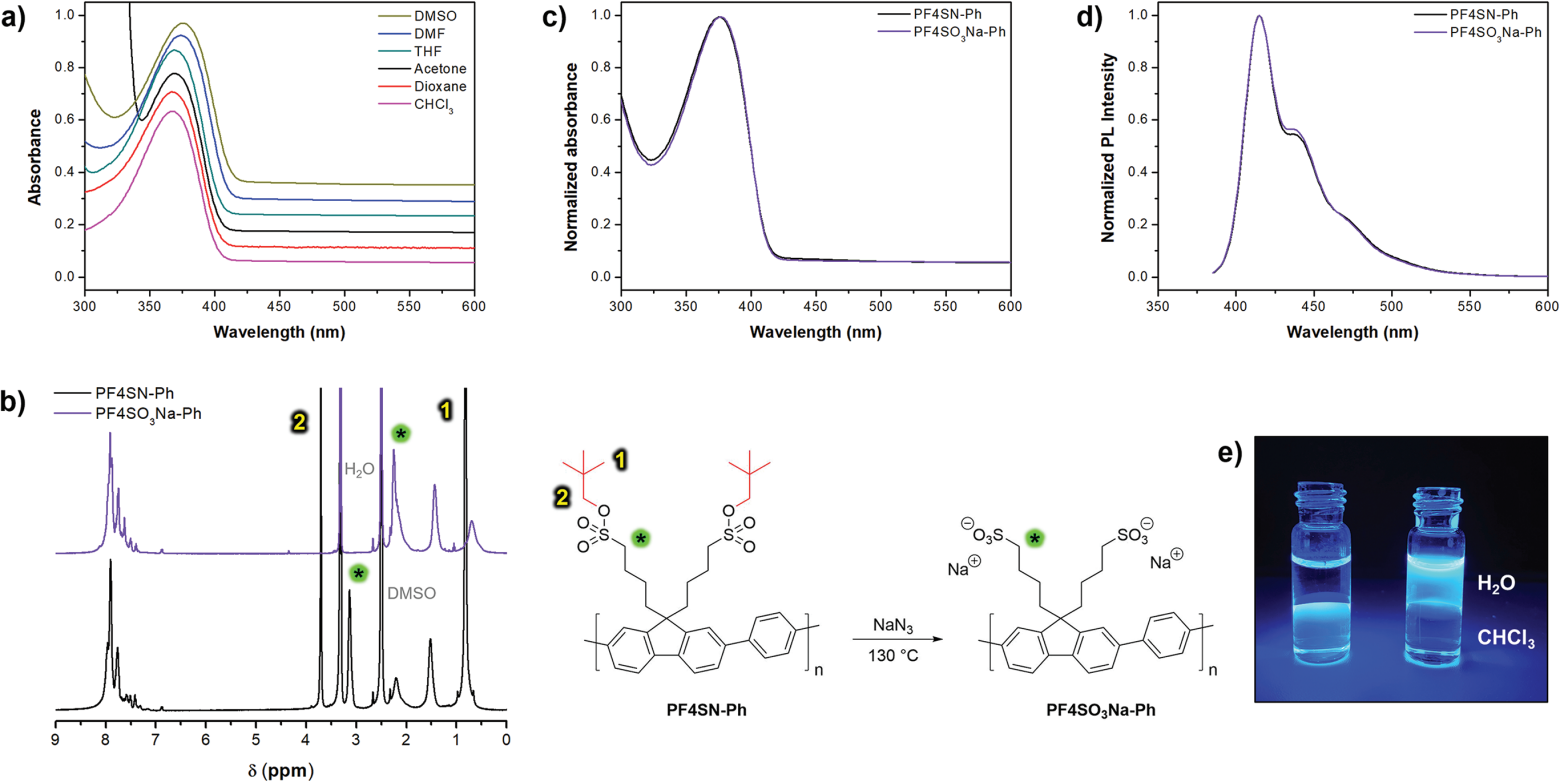
PF4SN‐Ph was used as model system for the strategy towards strong anionic CPEs. a) Stacked UV–Vis spectra of PF4SN‐Ph recorded in various polar organic solvents. b) ^1^H‐NMR spectra (DMSO‐d_6_) comparing the conjugated polymer before and after treatment with NaN_3_ (130°C), leading to quantitative removal of the neopentyl protecting groups (signals [1], [2], and [*]). c) UV–Vis and d) PL spectra of CPE/DMSO solutions before and after deprotection. e) Photograph of PF4SN‐Ph (left) and PF4SO_3_Na‐Ph (right) chloroform/water mixtures under UV illumination (365 nm).

A first interesting feature of PF4SN‐Ph is that the sulfonate esters render the copolymer soluble in various polar solvents, which is in large contrast to its *n*‐octyl functionalized hydrophobic relative PF8‐Ph (Figure S34, Supporting Information). Good ^1^H‐NMR spectra could be recorded in both CDCl_3_ and DMSO‐d_6_ (Figure S6, Supporting Information), and UV–Vis spectroscopy was performed in various solvents (**Figure** **1**a). A slight red‐shift was observed when moving from chloroform to more polar solvents like DMF ($\Delta \lambda_{\max}\approx 10$ nm); additional UV‐Vis and PL data can be found in the Supporting Information (Figure S19). Furthermore, both the absorption and emission properties are identical to PF8‐Ph synthesized in‐house (Figure S20, Supporting Information) and by others,^[^ ^36^ ^]^ thus implying that the sulfonate esters are sufficiently far away from the conjugated backbone to not have any impact. It is indeed well‐known that functional groups only affect the photophysical properties when they are directly attached to the aromatic backbone^[^ ^37^ ^]^ or are even part of the π‐conjugated system.^[^ ^38^ ^]^

Subsequently, deprotection of PF4SN‐Ph was carried out using the conditions previously developed for neopentyl‐protected poly(3‐sulfopropyl methacrylates), i.e., treatment of the copolymer with 3 eq. of the strong nucleophile NaN_3_ at 130 °C for 22 h; the neopentyl esters are stable towards acids, bases, and weak nucleophiles.^[^ ^32^ ^]^ Since both the protected and the sodium salt forms are soluble in DMSO, direct and accurate comparison can be performed by ^1^H‐NMR: complete disappearance of the characteristic neopentyl signals ([1], [2], and [*]) indicates quantitative deprotection and full conversion into PF4SO_3_Na‐Ph (Figure 1b; Figure S12a, Supporting Information). Besides that, FTIR provides qualitative confirmation of this success (Figure S13, Supporting Information). Deprotection can also be achieved at lower temperatures (100 °C), albeit requiring a longer reaction time (3 days). On the other hand, less than 20% of the neopentyl groups were removed in the absence of NaN_3_ (Figure S14, Supporting Information). A striking result is that the absorption and photoluminescence of PF4SN‐Ph and PF4SO_3_Na‐Ph are identical (Figure 1c,d), indicating NaN_3_ to be a sufficiently mild deprotecting agent—it does not affect the conjugated backbone and the direct conversion into the sodium sulfonate salt prevents acid‐induced doping of the polymer.^[^ ^20^, ^25^ ^]^ The molecular weight after deprotection was not further investigated by GPC, as polyelectrolytes are notoriously difficult to analyze using this technique and direct comparison with the precursor is no longer possible due to the loss of the protecting groups. The different solubilities of both copolymers are nicely demonstrated by illuminating a chloroform/water mixture with UV light (Figure 1e), although it should be noted that the amphiphilic structure causes the CPE to form aggregates in water^[^ ^1^, ^39^ ^]^ which is also confirmed by ^1^H‐NMR spectroscopy (Figure S12b). This effect is, however, hardly observed by UV‐Vis and PL spectroscopy; only a weak redshift was seen in PF4SO_3_Na‐Ph's PL spectrum (Figure S21, Supporting Information).

To further demonstrate the strength and robustness of our method, F4SN‐Br_2_ was copolymerized with other diboronic acid bis(pinacol) ester monomers to produce the alternating copolymers PF4SN‐BT, PF4SN‐F8, and PF4SN‐T (Scheme 1c). ^1^H‐NMR confirmed successful incorporation of the F4SN unit in the BT (benzothiadiazole) and F8 (dioctyl fluorene) copolymers without any deprotection occurring during the polycondensation (Figures S7 and S8, Supporting Information). Furthermore, decent molecular weights were obtained as evidenced by GPC (Figure S11, Supporting Information). Replacement of Ph by T (thiophene), on the other hand, resulted in an insoluble product that precipitated during the reaction; this problem could be resolved by moving to a different, water‐free, DMF‐based catalytic system that is also suitable for the synthesis of PF4SN‐Ph (Table S1, entry 6, Supporting Information). It turned out that PF4SN‐T (Figure S9, Supporting Information) is poorly soluble in the 1,4‐dioxane/*o*‐xylene solvent mixture that was used for the synthesis of the other copolymers, although it should not be excluded that the switch of solvent and catalyst suppressed side reactions, such as ring formation, as was previously observed for thiophene‐phenylene copolymers.^[^ ^40^ ^]^

Lastly, pure PF4SN homopolymer could be obtained by first converting F4SN‐Br_2_ into F4SN‐Bpin_2_ (Scheme 1a; Figure S4, Supporting Information),^[^ ^41^ ^]^ followed by copolymerization of the two under the same conditions as applied before (Figure S10, Supporting Information). Besides standard Suzuki polycondensation of AA and BB monomers, PF4SN could also be prepared via direct polymerization of F4SN‐Br_2_ using in‐situ boronation through the addition of bis(pinacolato)diboron. This approach, however, was accompanied by a lower yield and molecular weight (Table S2, entry 6, Supporting Information).^[^ ^42^, ^43^ ^]^ All macromolecular characteristics and reaction conditions of the PF4SN copolymers are listed in Table 1 and Table S2 (Supporting Information).

Before moving to the deprotection of the sulfonate groups, the thermal and structural properties of the hydrophobic precursors were first studied by TGA, DSC, and WAXS. In short, TGA indicated that the neopentyl groups are stable up to at least 210 °C (Figure S29, Supporting Information), where the polymers synthesized from larger comonomers are typically more stable due to the distance between the sulfonic esters and thermal deprotection proceeding via an in‐situ formed acid‐catalyzed process.^[^ ^31^ ^]^ The glass transition temperatures ($T_{g}$) of the copolymers range from 90 °C for the most flexible PF4SN‐F8 copolymer, up to 122 °C for most rigid PF4SN‐Ph copolymer (Figure S30, Supporting Information). These values are in the same region as alkylated polyfluorenes, although it should be noted that the $T_{g}s$ of polyfluorenes are strongly molecular weight‐dependent.^[^ ^44^ ^]^ Finally, regardless of the comonomer and the processing conditions, WAXS measurements indicated all PF4SN copolymers being non‐crystalline (Figures S31– S33, Supporting Information). This significantly deviating behavior from PF8 copolymers, in which various crystalline and liquid crystalline forms have been identified,^[^ ^45^ ^]^ can be attributed to the bulky nature of the neopentyl groups. More detailed analyses and discussions on both the thermal and structural properties can be found in the Supporting Information.

**Figure 2:**
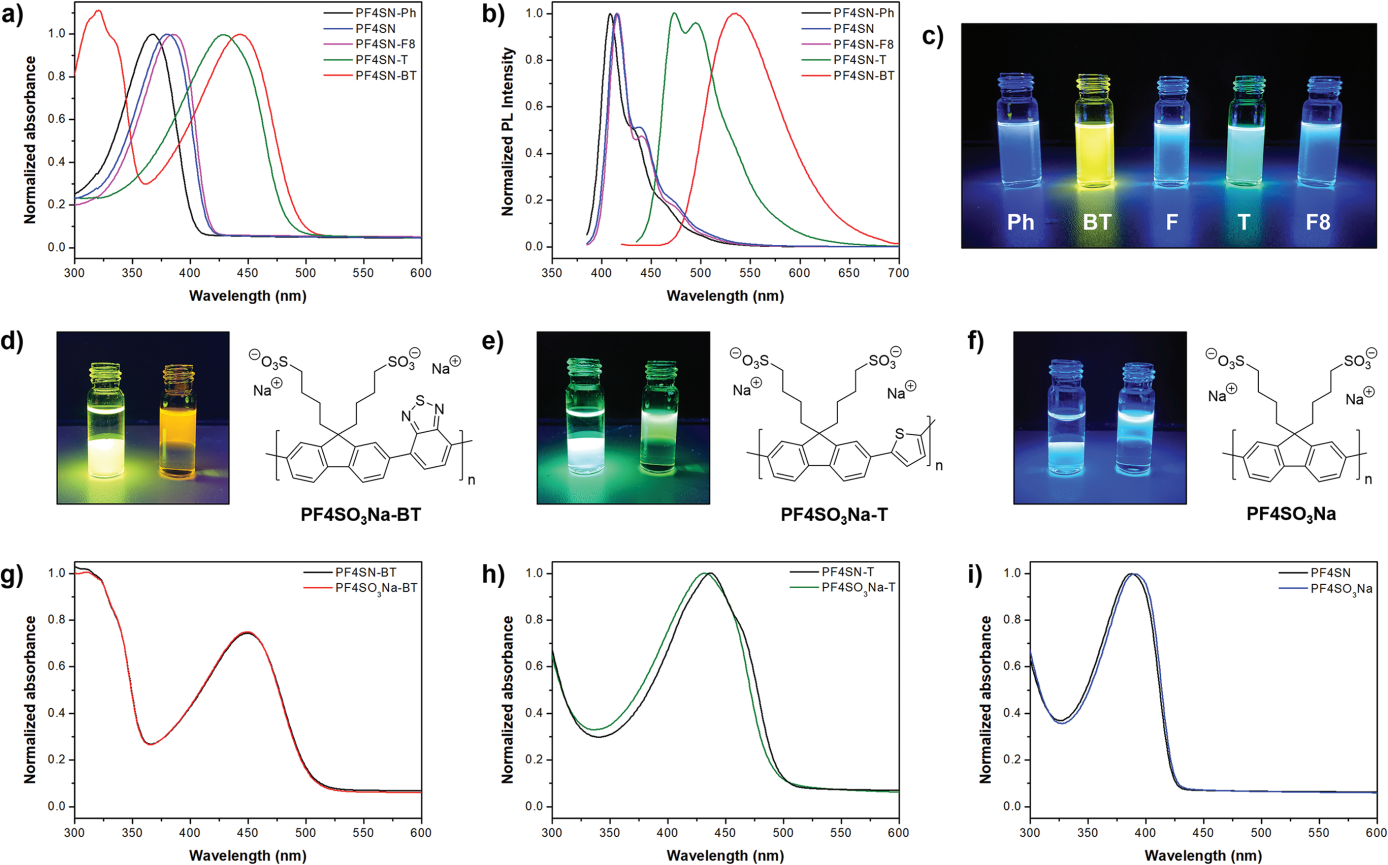
a) Normalized UV–Vis and b) PL spectra of chloroform‐based solutions of five different protected donor‐acceptor CPEs. c) Photograph of the CPE/chloroform solutions under UV irradiation (365 nm). From left to right: PF4SN‐Ph, PF4SN‐BT, PF4SN, PF4SN‐T, and PF4SN‐F8. d–f) Photographs of chloroform/water mixtures containing the PF4SN‐based CPEs before (left) and after (right) deprotection (UV illumination, 365 nm). g–i) Normalized UV–Vis spectra of BT and T copolymer‐ and F4SN homopolymer‐based CPE/DMSO solutions before and after deprotection.

The effect of the comonomer on the photophysical properties was investigated by studying chloroform‐based solutions using UV–Vis and PL spectroscopy (**Figure** **2**a–c). When F4SN was combined with thiophene and benzothiadiazole, the resulting donor‐acceptor structures of PF4SN‐T and PF4SN‐BT, respectively, resulted in red‐shifted spectra compared to PF4SN homopolymer.^[^ ^46^, ^47^ ^]^ Phenylene, on the other hand, has similar donating capabilities as fluorene, but the reduced planarity of the repeating unit led to somewhat blue‐shifted spectra. While having the same backbone, the small differences between PF4SN‐F8 and PF4SN can be attributed to a slight difference in chain conformation due the size and chemical nature of the side groups. Furthermore, the photophysical properties of all donor‐acceptor PF4SN copolymers are identical to their hydrophobic PF8 analogues, both synthesized in‐house (Figure S22, Supporting Information) and by others^[^ ^33^, ^48^, ^49^ ^]^ PF4SN‐F8 deserves special attention as the protected copolymer shows interesting UCST behavior in DMSO: it is only soluble on heating, becomes cloudy at room temperature, and ultimately forms a gel on standing. Such aggregation can be indirectly observed by UV–Vis spectroscopy and is presumably caused by the hydrophobic octyl groups, making DMSO only a moderately poor solvent for PF4SN‐F8,^[^ ^50^ ^]^ whereas all other PF4SN copolymers are fully soluble (Figures S23 and S24, Supporting Information). Finally, the synthesis method (conventional Suzuki polycondensation or via in‐situ boronation of F4SN‐Br_2_) does not affect the absorption characteristics of PF4SN homopolymer (Figure S25, Supporting Information).

Deprotection of the PF4SN copolymers was performed under the same conditions as applied earlier for PF4SN‐Ph. ^1^H‐NMR spectra (Figures S15– S18, Supporting Information) indicated quantitative removal of the neopentyl protecting groups, and resulted in conjugated polymers that are no longer soluble in common organic solvents. The formed PF4SO_3_Na CPEs are now fully soluble in both DMSO and water, except for the amphiphilic PF4SO_3_Na‐F8 copolymer (only soluble in DMSO). Photographs taken of chloroform/water mixtures under UV illumination nicely illustrate the different characters of the protected and deprotected polymers (Figure 2d–f). Here, the redshifted and weaker photoluminescence is caused by water being a rather poor solvent for the CPEs giving rise to aggregation due to their inherently amphiphilic nature, which was also evident from ^1^H‐NMR (Figures S15–S18, Supporting Information). Strong changes of the photoluminescence in water are indeed very common for CPEs; the absorption is usually less affected by this aggregation behavior (Figures S26 and S27, Supporting Information).^[^ ^1^ ^]^ Nevertheless, direct comparison of the protected and deprotected forms of the CPEs in the common and good solvent DMSO indicates that the photophysical properties are identical (Figures 2g–i; Figure S28, Supporting Information), thereby implying that NaN_3_ only acts as a nucleophile, does not affect the conjugated backbone, and that doping does not take place. These results confirm that the designed route is also suitable for the preparation of other fluorene‐based donor‐acceptor CPEs.

## 3 Conclusion

To conclude, the here presented method enables the synthesis of anionic fluorene donor‐acceptor CPEs via an elegant protection/deprotection approach. Isolation does not require tedious work‐up procedures, the use of a strong nucleophile results in quantitative removal of the protecting groups, while the photophysical properties remain unaffected and are identical to their hydrophobic PF8 relatives. Advantages over the commonly employed route towards anionic CPEs, that involves the polymerization of charged monomer species in water, include the suitability of existing protocols that have already been used for decades for hydrophobic monomers; complex catalytic systems optimized for aqueous polymerization are not needed.^[^ ^19^ ^]^ On top of that, the hydrophobic nature of the protected precursors allows facile macromolecular characterization using conventional techniques.

We believe that this elegant and non‐destructive method can be further expanded within the fluorene family using other monomers, such as carbazoles and cyclopentadithiophenes, to produce well‐defined narrow band gap CPEs. Furthermore, as a result of the high stability of the neopentyl protecting group, it is not unthinkable that this strategy can be easily translated to conjugated polymers that require other polymerization techniques, such as thiophenes and bithiophenes via direct arylation polymerization (DArP).^[^ ^51^ ^]^

In terms of applications, both the protected and deprotected forms can prove themselves useful: the anionic CPEs in chemical sensors, aqueous polyelectrolyte complexes, and as interlayer in electronic devices, whereas the amorphous protected precursors could be incorporated in devices that benefit from charge‐neutral conjugated polymers functionalized with polar side groups.^[^ ^52^ ^]^

## Conflict of Interest

The authors declare no conflict of interest.

## Author Contributions

A.H. contributed to the conceptualization, investigation, project administration, and the original draft writing. P.D. was involved in the investigation, and in reviewing and editing the manuscript. MK contributed to the writing by reviewing and editing the manuscript.

## Supporting information

Supporting Information

## Data Availability

The data that support the findings of this study are available from the corresponding author upon reasonable request.

## References

[1] H. Jiang , T. P ., J. R. Reynolds , K. S. Schanze , Angew. Chem. Int. Ed. 2009, 48, 4300. 10.1002/anie.200805456 19444838

[2] Q. Cui , G. C. Bazan , Acc. Chem. Res. 2018, 51, 202. 29240395 10.1021/acs.accounts.7b00501

[3] W. Ma , P. K. Iyer , X. Gong , B. Liu , D. Moses , G. C. Bazan , A. J. Heeger , Adv. Mater. 2005, 17, 274.

[4] J. Fang , B. H. Wallikewitz , F. Gao , G. Tu , C. Müller , G. Pace , R. H. Friend , W. T. S. Huck , J. Am. Chem. Soc. 2011, 133, 683. 21171591 10.1021/ja108541z

[5] J. Subbiah , V. D. Mitchell , N. K. C. Hui , D. J. Jones , W. W. H. Wong , Angew. Chem. Int. Ed. 2017, 56, 8431. 10.1002/anie.201612021 28256788

[6] M. Knaapila , R. C. Evans , V. M. Garamus , L. Almásy , N. K. Székely , A. Gutacker , U. Scherf , H. D. Burrows , Langmuir 2010, 26, 15634. 20822163 10.1021/la102591b

[7] A. R. Johnston , E. D. Minckler , M. C. J. Shockley , L. N. Matsushima , S. L. Perry , A. L. Ayzner , Angew. Chem. Int. Ed. 2022, 61, e202117759. 10.1002/anie.202117759 35229429

[8] S. P. O. Danielsen , T. Nguyen , G. H. Fredrickson , R. A. Segalman , ACS Macro Lett. 2019, 8, 88. 35619414 10.1021/acsmacrolett.8b00924

[9] M. L. Le , D. Rawlings , S. P. O. Danielsen , R. M. Kennard , M. L. Chabinyc , R. A. Segalman , ACS Macro Lett. 2021, 10, 1008. 35549124 10.1021/acsmacrolett.1c00354

[10] C. Dai , M. Panahandeh‐Fard , X. Gong , C. Xue , B. Liu , Sol. RRL 2019, 3, 1800255.

[11] J. Chen , Z. Fan , C. Zhang , H. Duan , L. Fan , ACS Appl. Polym. Mater. 2021, 3, 2088.

[12] H. Yuan , Y. Zhan , A. E. Rowan , C. Xing , P. H. J. Kouwer , Angew. Chem. Int. Ed. 2020, 59, 2720. 10.1002/anie.201910979 31917502

[13] A. Patil , Y. Ikenoue , F. Wudl , A. J. Heeger , J. Am. Chem. Soc. 1987, 109, 1858.

[14] S. Shi , F. Wudl , Macromolecules 1990, 23, 2119.

[15] C. Beaumont , S. Naqvi , M. Leclerc , Trends Chem. 2022, 4, 714.

[16] R. Richards , Y. Song , L. O'Connor , X. Wang , E. A. Dailing , A. E. Bragg , A. L. Ayzner , ACS Appl. Mater. Interfaces 2024, 16, 19995. 38289236 10.1021/acsami.3c14657 PMC11056932

[17] Z. B. Henson , Y. Zhang , T. Nguyen , J. H. Seo , G. C. Bazan , J. Am. Chem. Soc. 2013, 135, 4163. 23458636 10.1021/ja400140d

[18] C. Tan , M. R. Pinto , K. S. Schanze , Chem. Commun. 2002, 447. 10.1039/b109630c 12120534

[19] Y. Lin , H. Sun , H. Yang , Y. Lai , K. Hou , Y. Liu , Macromol. Rapid Commun. 2020, 41, 2000021. 10.1002/marc.202000021 32212226

[20] C. Mai , H. Zhou , Y. Zhang , Z. B. Henson , T. Nguyen , A. J. Heeger , G. C. Bazan , Angew. Chem. Int. Ed. 2013, 52, 12874. 10.1002/anie.201307667 24281883

[21] B. Ding , I. Jo , H. Yu , J. H. Kim , A. V. Marsh , E. Gutiérrez‐Fernández , N. Ramos , C. L. Rapley , M. Rimmele , Q. He , J. Martín , N. Gasparini , J. Nelson , M. Yoon , M. Heeney , Chem. Mater. 2023, 35, 3290. 37123107 10.1021/acs.chemmater.3c00327 PMC10134426

[22] J. C. Brendel , M. M. Schmidt , G. Hagen , R. Moos , M. Thelakkat , Chem. Mater. 2014, 26, 1992.

[23] K. Umezawa , T. Oshima , M. Yoshizawa‐Fujita , Y. Takeoka , M. Rikukawa , ACS Macro Lett. 2012, 1, 969. 35607053 10.1021/mz300290x

[24] P. Jagadesan , K. S. Schanze , Macromolecules 2019, 52, 3845.

[25] A. Mori , C. Kubota , K. Fujita , M. Hayashi , T. Ogura , T. Suzuki , K. Okano , M. Funahashi , M. Horie , Macromolecules 2020, 53, 1171.

[26] A. Kuwayama , S. Yamamoto , Y. Sakagami , M. Yamagishi , K. Okano , M. Horie , M. Funahashi , A. Mori , Polym. Chem. 2024, 15, 1635.

[27] C. Beaumont , T. Lemieux , S. Aivali , M. H. Sangachin , A. Gasonoo , T. M. St‐Pierre , M. Bélanger , S. Beaupré , G. C. Welch , M. Leclerc , ACS Macro Lett. 2024, 13, 1133. 39145595 10.1021/acsmacrolett.4c00397

[28] A. H. Hofman , R. Fokkink , M. Kamperman , Polym. Chem. 2019, 10, 6109. 10.1039/c9py00250b PMC8612725 34912475

[29] T. Pelras , A. H. Hofman , L. M. H. Germain , A. M. C. Maan , K. Loos , M. Kamperman , Macromolecules 2022, 55, 8795. 36245548 10.1021/acs.macromol.2c01487 PMC9558488

[30] M. Leclerc , J. Polym. Sci. Part A: Polym. Chem. 2001, 39, 2867.

[31] J. Kolomanska , P. Johnston , A. Gregori , I. F. Domínguez , H. Egelhaaf , S. Perrier , A. Rivaton , C. Dagron‐Lartigau , P. D. Topham , RSC Adv. 2015, 5, 66554.

[32] A. H. Hofman , M. Pedone , M. Kamperman , ACS Polym. Au 2022, 2, 169. 35698473 10.1021/acspolymersau.1c00044 PMC9185742

[33] R. Abbel , A. P. H. J. Schenning , E. W. Meijer , Macromolecules 2008, 41, 7497.

[34] C. Kulkarni , M. H. C. van Son , D. Di Nuzzo , S. C. J. Meskers , A. R. A. Palmans , E. W. Meijer , Chem. Mater. 2019, 31, 6633.

[35] M. Ranger , D. Rondeau , M. Leclerc , Macromolecules 1997, 30, 7686.

[36] G. Vamvounis , M. Fuhrer , K. Keller , L. Willig , A. Koizumi , H. Hu , M. Gao , T. D. M. Bell , Eur. Polym. J. 2019, 119, 551.

[37] M. Rimmele , F. Glöcklhofer , M. Heeney , Mater. Horiz. 2022, 9, 2678. 35983884 10.1039/d2mh00519k PMC9620492

[38] T. P. Voortman , R. C. Chiechi , ACS Appl. Mater. Interfaces 2015, 7, 28006. 25723354 10.1021/acsami.5b00564

[39] H. D. Burrows , M. J. Tapia , S. M. Fonseca , A. J. M. Valente , V. M. M. Lobo , L. L. G. Justino , S. Qiu , S. Pradhan , U. Scherf , N. Chattopadhyay , M. Knaapila , V. M. Garamus , ACS Appl. Mater. Interfaces 2009, 1, 864. 20356013 10.1021/am800267n

[40] M. Jayakannan , J. L. J. van Dongen , R. A. J. Janssen , Macromolecules 2001, 34, 5386.

[41] T. Ishiyama , M. Murata , N. Miyaura , J. Org. Chem. 1995, 60, 7508.

[42] A. Izumi , R. Nomura , T. Masuda , Chem. Lett. 2000, 29, 728.

[43] F. Brouwer , J. Alma , H. Valkenier , T. P. Voortman , J. Hillebrand , R. C. Chiechi , J. C. Hummelen , J. Mater. Chem. 2011, 21, 1582.

[44] C. Müller , Chem. Mater. 2015, 27, 2740.

[45] M. Knaapila , M. J. Winokur , Adv. Polym. Sci. 2008, 212, 227.

[46] Z. Zhang , J. Wang , J. Mater. Chem. 2012, 22, 4178.

[47] K. Müllen , U. Scherf , Macromol. Chem. Phys. 2023, 224, 2200337.

[48] S. Inagi , S. Hayashi , K. Hosaka , T. Fuchigami , Macromolecules 2009, 42, 3881.

[49] I. R. Grova , A. G. Macedo , L. S. Roman , L. Akcelrud , Eur. Polym. J. 2013, 49, 3539.

[50] D. D. C. Bradley , M. Grell , X. Long , H. Mellor , A. Grice , M. Inbasekaran , E. P. Woo , Proc. SPIE 1997, 3145, 254.

[51] J. Kimpel , Y. Kim , J. Asatryan , J. Martín , R. Kroon , C. Müller , Chem. Sci. 2024, 15, 7679. 38784738 10.1039/d4sc01430h PMC11110131

[52] Y. Zhang , G. Ye , T. P. A. van der Pol , J. Dong , E. R. W. van Doremaele , I. Krauhausen , Y. Liu , P. Gkoupidenis , G. Portale , J. Song , R. C. Chiechi , Y. van de Burgt , Adv. Funct. Mater. 2022, 32, 2201593.

