# Supplementary material for: Strong Anionic Fluorene Donor–Acceptor Copolyelectrolytes from Protected Hydrophobic Precursors
Source: Macromol Rapid Commun. 2025 Jan 2;46(5):2400925. doi: 10.1002/marc.202400925 (PMC11884233; doi:10.1002/marc.202400925)
Supplement: Supplementary file 1 — Supporting Information [file MARC-46-2400925-s001.pdf]

Macromolecular Rapid Communications

Supporting Information

for *Macromol. Rapid Commun.*, DOI 10.1002/marc.202400925

Strong Anionic Fluorene Donor–Acceptor Copolyelectrolytes from Protected Hydrophobic Precursors

Anton H. Hofman, Peter Dijkstra and Marleen Kamperman*

Supporting Information for

Strong Anionic Fluorene Donor-Acceptor Copolyelectrolytes from Protected Hydrophobic Precursors

Anton H. Hofman*, Peter Dijkstra, and Marleen Kamperman

Polymer Science, Zernike Institute for Advanced Materials,
University of Groningen, Nijenborgh 3, 9747 AG Groningen, The Netherlands

*a.h.hofman@rug.nl

1 Experimental section

1.1 Materials

Fluorene (> 95%), sodium hydride (NaH, 60%, dispersion in paraffin liquid), triethylamine (98%), bis(pinacolato)diboron (BiPi, > 99%), tetrakis(triphenylphosphine) palladium(0) ($Pd(PPh_3)_4$, > 97.0%), and silica (60 μm, for column chromatography) were purchased from TCI Europe N.V. and used without further purification. Anhydrous *N,N*-dimethylformamide (DMF, 99.8%), anhydrous 1,4-dioxane (99.8%), 1,4-butane sultone (> 99%), neopentyl alcohol (> 99.5%), potassium acetate (≥ 99.0%), [1,1'-bis(diphenyl phosphino)ferrocene]dichloropalladium(II) ($Pd(dppf)Cl_2$), Aliquat 336®, diethyldithiocarbamic acid diethylammonium salt (97%), 1,4-benzenediboronic acid bis(pinacol) ester ($Ph\text{-}Bpin_2$, 97%), 9,9-dioctyl-9H-fluorene-2,7-diboronic acid bis(pinacol) ester ($F8\text{-}Bpin_2$, 96%), 2,1,3-benzothiadiazole-4,7-bis(boronic acid pinacol ester) ($BT\text{-}Bpin_2$, 95%), thiophene-2,5-diboronic acid bis(pinacol) ester ($T\text{-}Bpin_2$, 97%), sodium azide (NaN_3, ≥ 99.5%), anhydrous magnesium sulfate ($MgSO_4$, > 99.5%), sodium bicarbonate ($NaHCO_3$, > 98%), water-free sodium thiosulfate ($Na_2S_2O_3$, > 98%), potassium carbonate (K_2CO_3, 99%), potassium phosphate (K_3PO_4, > 98%) were obtained from Sigma-Aldrich and used as received. Oxalyl chloride (99%) was acquired from Acros Organics and *o*-xylene (99%) from Alfa Aesar. All other analytical grade solvents were obtained from Macron Fine Chemicals. *N*-bromosuccinimide (NBS, TCI Europe N.V., 98%) was recrystallized from DI water prior to use. Thin-layer chromatography (TLC) was performed on Macherey-Nagel Alugram UV254 indicator/silica-coated aluminum sheets.

The hydrophobic alkylated polyfluorenes (i.e., PF8, PF8-Ph, PF8-BT, and PF8-T – **Figure S34**) used for comparative studies were synthesized via standard, non-optimized Suzuki polycondensation from unpurified commercially available monomers. The results are summarized in **Table S5**.

1.2 Synthesis of protected monomers

Sodium 4,4'-(9H-fluorene-9,9-diyl)bis(butane-1-sulfonate) ($F4SO_3Na$)

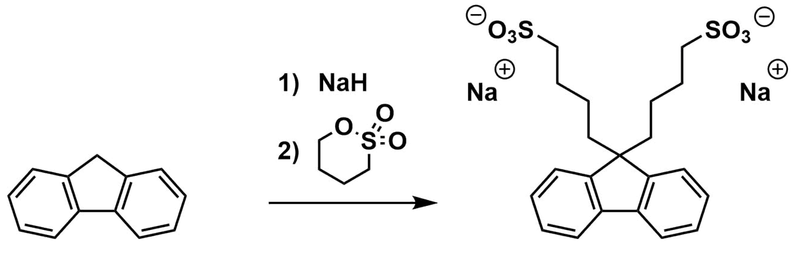

Fluorene was functionalized with sulfonated butyl groups through ring opening of 1,4-butane sultone.[1] First, in a 250 ml three-neck round-bottom flask, 4.06 g (24.4 mmol; 1.0 eq.) fluorene was dissolved in 70 ml DMSO under an argon atmosphere. Then 3.95 g (98.8 mmol; 4.0 eq.) NaH (60 wt% in mineral oil) was added portionwise as a solid.[2] The colorless solution turned orange instantaneously which was accompanied by significant amounts of gas release. 15 min after complete addition of NaH, the reaction mixture was cooled with a water bath and 8.03 g (59.0 mmol; 2.4 eq.) 1,4-butane sultone was added dropwise as a 10 ml solution in DMSO. The now red solution continued bubbling,

turned purple over time, and was allowed to stir at room temperature overnight. The next morning, the still purple solution was precipitated into 600 ml acetone, filtered using a glass filter funnel (pore size 4) and washed with copious amounts of acetone in order to remove unreacted 1,4-butane sultone. The obtained orange slurry was redispersed in 300 ml absolute ethanol and stirred for 4 h to dissolve any remaining base. The slurry was filtered (glass filter funnel, pore size 4), washed with ethanol, then neutralized with ethanol containing a few drops concentrated hydrochloric acid, more ethanol, acetone, and finally some pentane. The powder was transferred to a beaker and dried in a vacuum oven overnight (40 °C) and shortly dried in a regular oven at 130 °C. F4SO_3Na was obtained as an off-white powder (9.2 g) and used without further purification in the next step (yield: 78%).

^{1}H-NMR (D_2O, 400 MHz): δ (ppm) = 7.83 (d, 2 H; Ar-H), 7.52 (d, 2 H; Ar-H), 7.41 (m, 4 H; Ar-H), 2.58 (t, 4 H; CH_2), 2.08 (t, 4 H; CH_2), 1.45 (quint, 4 H; CH_2), and 0.62 (quint, 4 H; CH_2) (**Figure S1**).

Dineopentyl 4,4'-(9H-fluorene-9,9-diyl)bis(butane-1-sulfonate) (F4SN)

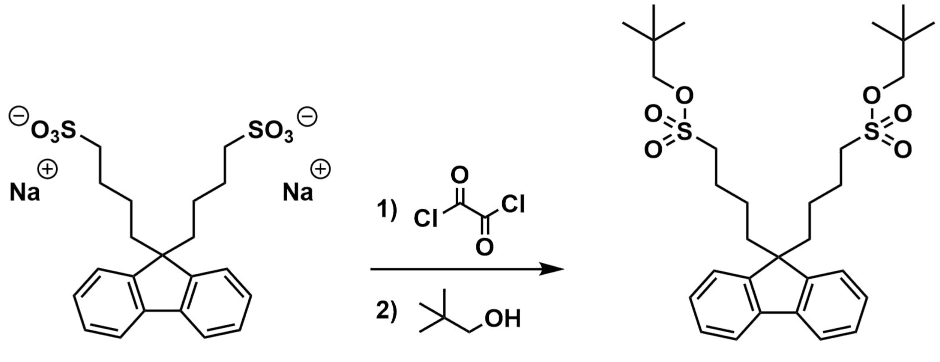

The sulfonate functional groups of F4SO_3Na were protected with neopentyl esters by using the strategy previously reported for 3-sulfopropyl methacrylates,[3,4] although some modifications were necessary in order to functionalize both groups and increase the yields.

A 100 ml three-neck round-bottom flask was charged with 4.50 g (9.33 mmol; 1.0 eq.) F4SO_3Na, and vacuum and heat were applied for approximately 30 min to remove the last traces of solvent. After cooling down to room temperature, the sodium salt was dispersed in 14 ml anhydrous DMF. Then the flask was further cooled using an ice bath (0 °C), and 2.91 g (22.9 mmol; 2.5 eq.) oxalyl chloride in 7.5 ml dichloromethane was added dropwise via a glass pipette under an argon outflow. After completion, the cloudy reaction mixture was stirred for 1 h at 0 °C and another 2 h at room temperature.

In another 100 ml three-neck round-bottom flask that was equipped with a dropping funnel, a solution of neopentyl alcohol (2.18 g; 24.7 mmol; 2.6 eq.) and triethylamine (3.81 g; 37.7 mmol; 4.0 eq.) in 15 ml dichloromethane was prepared under an argon atmosphere, and cooled to 0 °C. The yellow/brown F4SO_2Cl/NaCl suspension was carefully transferred to the dropping funnel and slowly added over a period of about 20 min. The orange suspension was stirred at 0 °C for 1 h, and subsequently allowed to warm up overnight. The next morning the reaction was quenched with 200 ml DI water, extracted with three portions diethyl ether (150 ml each), and the combined organic fractions were

washed with 200 ml water. After being dried over $MgSO_4$, the solution was concentrated in vacuo to give the crude product as a dark orange oil. Purification was accomplished by silica gel column chromatography using hexane/ethyl acetate (4/1) as eluent ($R_f \approx 0.5$). F4SN was obtained as a clear colorless oil (3.55 g) that could be crystallized by evaporation from a hexane/diethyl ether mixture. The white powder was stored in the fridge until further use. Yield: 65.8%.

^{1}H-NMR ($CDCl_3$, 400 MHz): δ (ppm) = 7.70 (d, 2 H; Ar-H), 7.32 (m, 6 H; Ar-H), 3.72 (s, 4 H; CH_2), 2.80 (t, 4 H; CH_2), 2.03 (t, 4 H; CH_2), 1.60 (quint, 4 H; CH_2), 0.90 (s, 18 H; CH_3), and 0.67 (quint, 4 H; CH_2) (**Figure S2a**).

^{13}C-NMR ($CDCl_3$, 100 MHz): δ (ppm) = 22.8, 23.8, 26.2, 31.8, 40.0, 50.0, 54.6, 78.5, 120.1, 122.7, 127.5, 127.6, 141.2, and 149.1 (**Figure S2b**).

EA: C 64.03% (calc. 64.33%), H 8.28% (calc. 8.01%), N 0.00% (calc. 0.00%), S 11.29% (calc. 11.08%).

Dineopentyl 4,4'-(2,7-dibromo-9H-fluorene-9,9-diyl)bis(butane-1-sulfonate) (F4SN-Br_2)

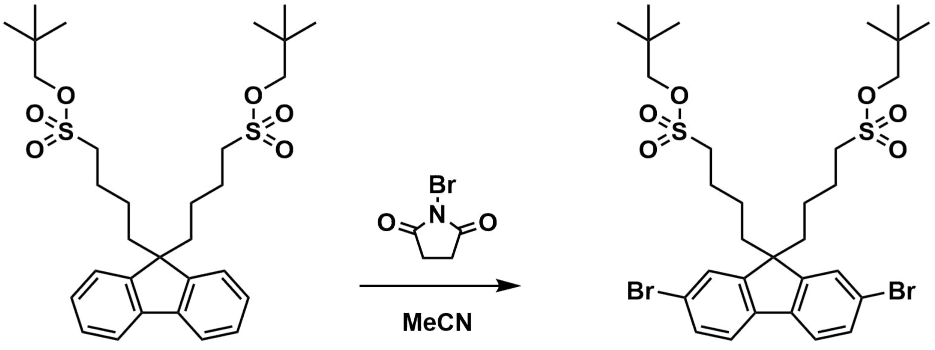

F4SN-Br_2 was synthesized through bromination of F4SN using an excess N-bromosuccinimide (NBS). The reaction could only be driven to completion by performing the bromination at elevated temperatures. It should be noted that solvents like THF, DMF, NMP, and toluene should be avoided under such conditions.[5]

In a 100 ml two-neck round-bottom flask under an argon atmosphere, 3.36 g (5.80 mmol, 1.0 eq.) F4SN was dissolved in 13 ml acetonitrile. Then 2.71 g (15.2 mmol; 2.6 eq.) NBS was added in portions over a period of about 10 minutes; the clear and colourless reaction mixture turned orange rapidly. Upon complete addition, the solution was heated to 60 °C and stirred for 20 h. Progress of the bromination reaction was monitored by TLC and ^{1}H-NMR. After cooling down to room temperature, the orange solution was diluted with 150 ml DI water and extracted three times with diethyl ether (150 ml each). Next, the combined organic fractions were sequentially washed with 150 ml 5 wt% $NaHCO_{3(aq)}$ and 5 wt% $Na_2S_2O_{3(aq)}$ causing the ether solution to become colourless. Subsequently it was dried over $MgSO_4$, filtered, and the organic solvents were removed in vacuo to afford crude F4SN-Br_2 as a faint yellow oil. The product was purified by silica gel column chromatography using hexane/ethyl acetate (3/1) as eluent ($R_f \approx 0.5$). F4SN-Br_2 was obtained as a white solid after evaporation from a hexane/diethyl ether mixture.

Traces of solvent were removed by drying the powder under high vacuum for approximately 30 min, and it was stored in the fridge until further use. Yield: 3.32 g (77.6%).

^{1}H-NMR ($CDCl_3$, 400 MHz): δ (ppm) = 7.54 – 7.47 (m, 4 H; Ar-H), 7.43 (s, 2 H; Ar-H), 3.75 (s, 4 H; CH_2), 2.84 (t, 4 H; CH_2), 1.99 (t, 4 H; CH_2), 1.64 (quint, 4 H; CH_2), 0.92 (s, 18 H; CH_3), and 0.68 (quint, 4 H; CH_2) (**Figure S3a**).

^{13}C-NMR ($CDCl_3$, 100 MHz): δ (ppm) = 22.7, 23.8, 26.2, 31.8, 39.8, 49.9, 55.2, 78.5, 121.6, 122.1, 126.1, 131.0, 139.1, and 151.1 (**Figure S3b**).

EA: C 50.33% (calc. 50.55%), H 5.99% (calc. 6.02%), N 0.00% (calc. 0.00%), S 8.80% (calc. 8.70%).

Dineopentyl 4,4'-(2,7-bis(4,4,5,5-tetramethyl-1,3,2-dioxaborolan-2-yl)-9H-fluorene-9,9-diyl) bis(butane-1-sulfonate) (F4SN-$Bpin_2$)

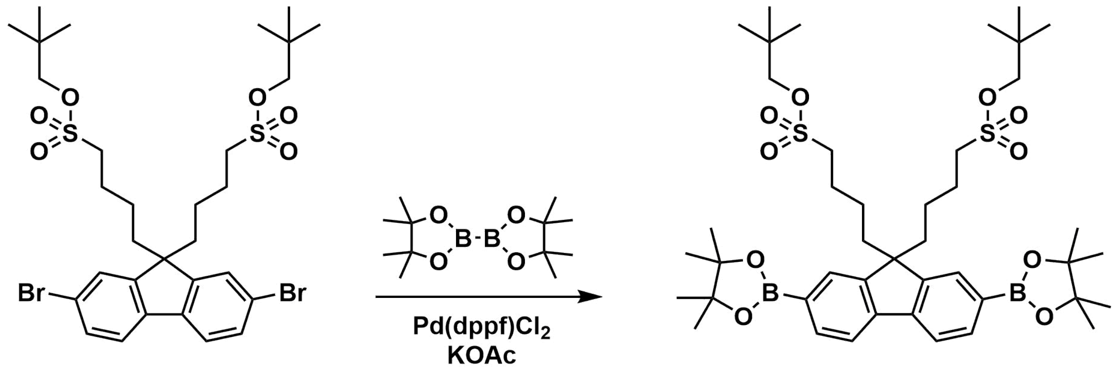

Boronic acid pinacol esters were installed on F4SN via a Miyaura borylation reaction using a modified literature procedure.[6,7] In a 50 ml two-neck round-bottom flask under an argon atmosphere, 1.25 g F4SN-Br_2 (1.70 mmol; 1.0 eq.), 962 mg bis(pinacolato)diboron (3.79 mmol; 2.2 eq.), 701 mg potassium acetate (7.14 mmol; 4.2 eq.), and 61.9 mg Pd(dppf)Cl_2 (0.0846 mmol; 0.050 eq.) were dissolved in 8.5 ml degassed DMSO. The dark solution was stirred at 80 °C for 21 h. After cooling down to room temperature, the reaction mixture was diluted with 100 ml DI water and extracted with three portions diethyl ether (100 ml each). The combined organic fractions were washed once more with water (100 ml), subsequently dried over $MgSO_4$, filtered, and concentrated using a rotary evaporator resulting in a brown solid. The crude product was purified by silica gel chromatography using hexane/acetone (3/1) as eluent ($R_f \approx 0.4$) to give F4SN-$Bpin_2$ as a white solid. Traces of solvent were removed by drying the powder under high vacuum for approximately 1 h. It was stored in the fridge until further use. Yield: 0.88 g (62%).

^{1}H-NMR ($CDCl_3$, 400 MHz): δ (ppm) = 7.83 (d, 2 H; Ar-H), 7.72 (m, 4 H; Ar-H), 3.72 (s, 4 H; CH_2), 2.78 (t, 4 H; CH_2), 2.07 (t, 4 H; CH_2), 1.60 (quint, 4 H; CH_2), 1.40 (s, 24 H; CH_3), 0.90 (s, 18 H; CH_3), and 0.59 (quint, 4 H; CH_2) (**Figure S4a**).

^{13}C-NMR ($CDCl_3$, 100 MHz): δ (ppm) = 22.7, 23.8, 25.1, 26.2, 31.8, 39.8, 50.1, 54.8, 78.5, 84.1, 119.8, 128.6, 134.4, 143.9, and 149.0 (**Figure S4b**).

EA: C 62.35% (calc. 62.17%), H 8.19% (calc. 8.25%), N 0.15% (calc. 0.00%), S 7.53% (calc. 7.72%).

1.3 Polymerizations

Suzuki polycondensation (method A)

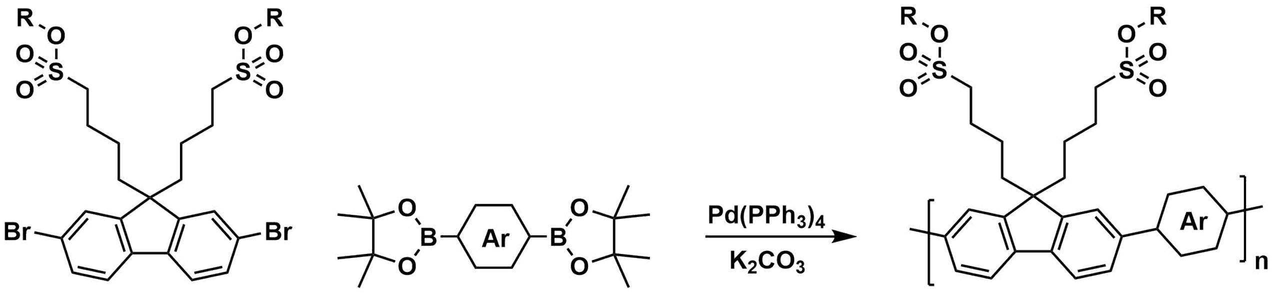

The majority of the PF4SN CPEs were synthesized following a conventional Suzuki polycondensation procedure. An example for PF4SN-Ph is given below (**Table S2, entry 1**).

A 25 ml two-neck round-bottom flask was placed under an argon atmosphere, and was subsequently charged with 299 mg F4SN-Br_2 (0.405 mmol; 1.0 eq.), 134 mg 1,4-benzenediboronic acid bis(pinacol) ester (Ph-$Bpin_2$) (0.406 mmol; 1.0 eq.) and 23.7 mg $Pd(PPh_3)_4$ (0.0205 mmol; 0.051 eq.). The solids were dissolved in 3.2 ml of a degassed 1,4-dioxane/*o*-xylene (1/1) mixture containing 10 mg ml^{-1} Aliquat 336. Then 1.0 ml of a degassed aqueous 4.0 M K_2CO_3 solution was added. The clear yellow/colourless two-layer system was reacted at 90 °C for 24 h while stirring vigorously; the organic layer darkened over time. After cooling down to room temperature, the reaction mixture was diluted with 50 ml 1.0 M HCl and extracted with three portions chloroform (50 ml each). The combined organic fractions were dried over $MgSO_4$, filtered, and concentrated in vacuo to result in a dark oil that was redissolved in 3 ml THF. 88 mg (0.40 mmol) Pd scavenger (diethyldithiocarbamic acid diethylammonium salt) was added and the dark solution was stirred at room temperature for 2 h.[8,9] The polymer was precipitated into 80 ml ethanol, filtered using a glass filter funnel (pore size 4), sequentially washed with ethanol and methanol, and dried in a vacuum oven overnight (40 °C) to afford PF4SN-Ph as an off-white powder. Yield: 0.22 g (83%).

Other CPEs were obtained by replacing the Ph-$Bpin_2$ comonomer for BT-$Bpin_2$, F8-$Bpin_2$, and F4SN-$Bpin_2$ to give PF4SN-BT, PF4SN-F8 and PF4SN, respectively (**Table S2, entries 2, 3, and 5**). All protected CPEs were stored in the fridge until further use.

Heterogeneous Suzuki polycondensation (method B)

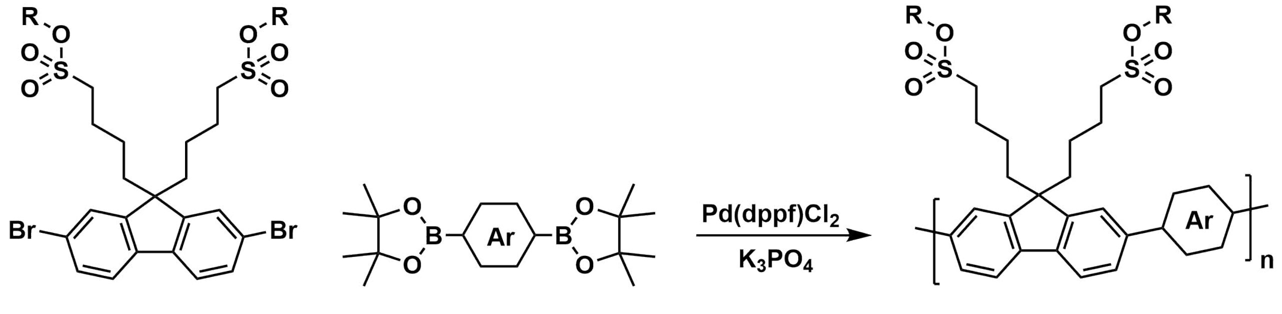

Because of solubility issues when using the biphasic liquid approach (route A), PF4SN-T was synthesized in a heterogeneous medium, using DMF as solvent and without the addition of water (**Table S2, entry 4**).

To a 25 ml two-neck round-bottom flask were added 223 mg F4SN-Br_2 (0.303 mmol; 1.0 eq.), 101 mg thiophene-2,5-diboronic acid bis(pinacol) ester (T-$Bpin_2$) (0.302 mmol; 1.0 eq.), 11.1 mg Pd(dppf)Cl_2 (0.0152 mmol; 0.050 eq.), and 362 mg K_3PO_4 (1.71 mmol; 5.6 eq.). The setup was evacuated and placed under an argon atmosphere. Next, 4.0 ml degassed DMF containing 7.8 mg ml^{-1} Aliquat 336 was added; the solution turned dark yellow within minutes. The Suzuki polycondensation was carried out at 80 °C for 24 h while stirring vigorously. Next, the dark solution was cooled down to room temperature, diluted with chloroform and 50 ml 1.0 M HCl, extracted with three portions of chloroform (50 ml each), dried over $MgSO_4$, filtered, and concentrated in vacuo. The resulting dark oil was diluted with 2 ml THF and after addition of 79 mg (0.36 mmol) Pd scavenger (diethyldithiocarbamic acid diethylammonium salt) the dark solution was stirred for 1.5 h at room temperature. The polymer was precipitated into 70 ml ethanol, filtered using a glass filter funnel (pore size 4), washed with ethanol and methanol, and finally dried in a vacuum oven overnight (40 °C) to give PF4SN-T as a dark green, almost black powder that was stored in the fridge until further use. Yield: 0.15 g (75%).

Following this route, PF4SN-Ph could also be successfully synthesized by replacing T-$Bpin_2$ for Ph-$Bpin_2$ (**Table S1, entry 6**).

PF4SN homopolymer - BiPi route (method C)

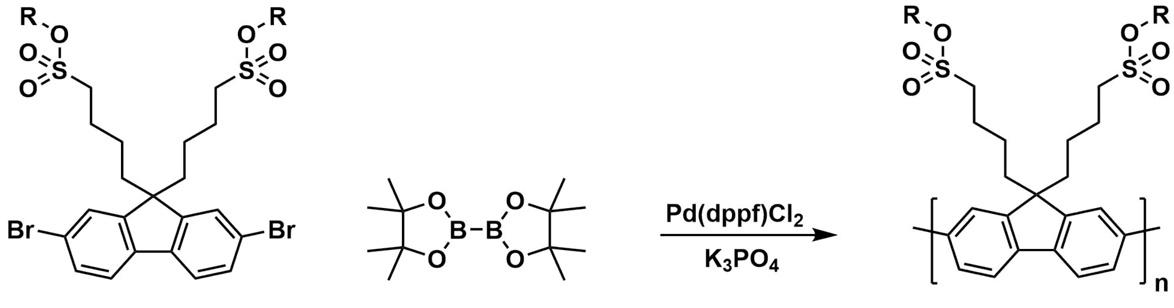

Instead of copolymerization of F4SN-Br_2 and F4SN-$Bpin_2$, PF4SN homopolymer could also be directly obtained through polymerization of F4SN-Br_2 in the presence of bis(pinacolato)diboron (BiPi) (**Table S2, entry 6**).[10–12]

A 25 ml two-neck round-bottom flask was charged with 315 mg F4SN-Br_2 (0.427 mmol; 1.0 eq.), 108 mg BiPi (0.426 mmol; 1.0 eq.), 16.2 mg Pd(dppf)Cl_2 (0.0221 mmol; 0.052 eq.), and 469 mg K_3PO_4 (2.21 mmol; 5.2 eq.). The setup was evacuated and placed under an argon atmosphere. Then 4.0 ml degassed DMF containing 8.1 mg ml^{-1} Aliquat 336 was added; the resulting solution turned brown within minutes. The polymerization was performed at 80 °C for 24 h under vigorous stirring. After cooling down to room temperature, the dark solution was diluted with chloroform and 50 ml 1.0 M HCl, extracted with three portions of chloroform (50 ml each), dried over $MgSO_4$, filtered, and concentrated under reduced pressure. The obtained viscous dark brown oil was diluted with 2.0 ml THF, 98 mg (0.44 mmol) Pd scavenger (diethyldithiocarbamic acid diethylammonium salt) was added and the resulting solution was allowed to stir for 2 h at room temperature. The polymer was precipitated into 60 ml of an ethanol/pentane mixture (1/1), filtered using a glass filter funnel (pore size 4), sequentially washed with more of this 1/1 mixture and methanol, and finally dried in a vacuum oven overnight (40 °C) to give PF4SN as a light brown powder. It was stored in the fridge until further use. Yield: 0.11 g (45%).

1.4 Deprotection

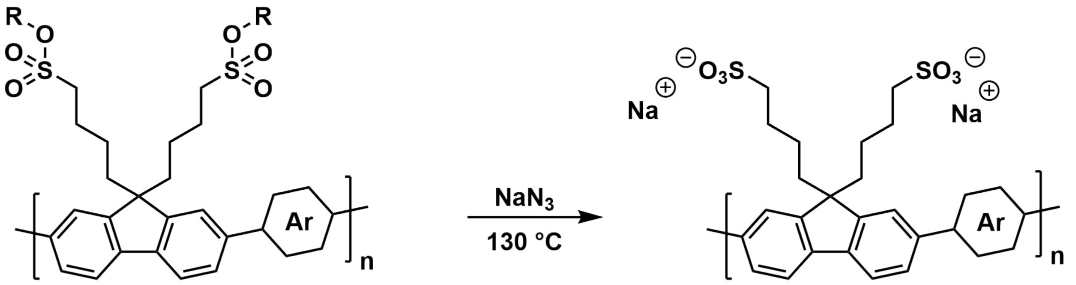

The neopentyl protecting groups were quantitatively removed by treating the protected CPEs with sodium azide (3 eq. NaN_3 vs RSO_3Neo). An example for PF4SN-Ph is given below; the other protected CPEs (PF4SN-BT, PF4SN-F8, PF4SN-T and PF4SN) were converted into strong polyanions by applying the same strategy.

A 20 ml screw cap vial was charged with 73.9 mg PF4SN-Ph (0.226 mmol RSO_3Neo groups; 1.0 eq.) and 48.1 mg NaN_3 (0.740 mmol; 3.3 eq.). Then 1.2 ml DMSO was added, the vial was closed, and the mixture was stirred for 22 h at 130 °C; the dispersion turned into a homogeneous solution within minutes. After cooling down to room temperature, the cloudy reaction mixture was diluted with 1 ml DMSO and precipitated into 60 ml ethanol/pentane (1/1). The fine white precipitate was collected by centrifugation. Next, the obtained polymer cake was resuspended in approximately 50 ml fresh ethanol/pentane (1/1) and centrifuged again; this washing procedure was repeated two more times to remove the excess NaN_3. The CPE was finally washed with pentane and dried in vacuo to obtain PF4SO_3Na-Ph as a fine white powder. Yield: 60 mg (95%).

1.5 Characterization

^1H and ^{13}C nuclear magnetic resonance (NMR) spectra were recorded at room temperature on either a Varian VXR-400 or Agilent 400-MR spectrometer. A pulse width of 45 μs, spectral width of 12/-2 ppm, recycle delay of 1 s and 32 scans were applied for ^1H measurements. All spectra were analyzed with MestReNova software. Chemical shifts (δ) are reported in parts per million (ppm) relative to the residual solvent peak ($CDCl_3$, DMSO-d_6, or D_2O).

Fourier transform infrared spectroscopy (FTIR) was carried out on a Bruker Vertex 70 instrument at room temperature in attenuated total reflection (ATR) mode. Spectra were acquired in the range of 4000 – 500 cm^{-1} and the data was analyzed using OPUS software.

The molecular weights (M_n) and their distribution (Đ) were measured by gel permeation chromatography (GPC) using a triple detection setup, consisting of a Malvern Viscotek Dual Detector and a Schambeck RI 2012 refractive index detector. Chloroform (CHROMASOLV, for HPLC, $>$ 99.8%, amylene stabilized) was used as eluent. Separation was carried out by utilizing two PLgel 5 μm MIXED-C 300 mm columns from Agilent Technologies at a flow rate of 0.5 ml min^{-1} and a temperature of 35 °C. Narrow polystyrene standards (Agilent and Polymer Laboratories, M_n ranging from 645 to 3,001,000 g mol^{-1}) were used for calibration of the system. Samples (1 – 2 mg ml^{-1}) were filtered over a 0.2 μm PTFE filter prior to injection. Molecular weights of the protected conjugated polyelectrolytes were calculated by applying conventional calibration using Viscotek OmniSEC software version 5.0.

Differential scanning calorimetry (DSC) measurements were conducted on a TA Instruments DSC Q1000 under a nitrogen atmosphere. The samples were heated to 170 °C and cooled back to -30 °C at a rate of 10 °C min^{-1} with the second heating cycle being used for analysis. Glass transition temperatures (T_g) were determined by using TA Instruments Universal Analysis software. Multiple heating cycles did not result in an increase or decrease of the T_g, indicating sufficient thermal stability of the protecting groups throughout the measurement.

Thermogravimetric analysis (TGA) was performed on a TA Instruments TGA 5500 under a continuous nitrogen flow where samples were heated from 30 to 700 °C at a rate of 10 °C min^{-1}. Data acquisition and analysis were done by using TA Instruments TRIOS software. The onset temperature of degradation (T_{onset}) is the extrapolated onset (i.e., the intersection of the two tangents), the peak temperature (T_{peak}) is defined as the temperature at which the weight loss rate reaches its maximum (inflection point, $\partial^2 m/\partial T^2 = 0$), and the weight loss ($Wt$) is calculated from the end point of the degradation process of interest, which is defined as the point at which the weight loss rate reaches its minimum (inflection point, $\partial^2 m/\partial T^2 = 0$). Results are summarized in **Table S3**.

UV-Vis spectroscopy was carried on an Analytik Jena Specord 210 Plus spectrophotometer using 10 $\times$ 10 mm quartz cuvettes. Stock solutions (1.0 mg ml^{-1}) were prepared and diluted until the maximum absorbance dropped below 1.0. This typically corresponded to a concentration of approximately 0.05 mg ml^{-1}, depending on the polymer and solvent. Spectra were recorded at a speed of 10 nm s^{-1} with 1 nm intervals and an integration time of 0.1 s. The acquired data was analyzed using Aspect UV software.

Fluorescence spectra were recorded on a JASCO FP-7200 spectrofluorometer. The same stock solutions as used earlier for UV-Vis spectroscopy were diluted in 10×10 mm quartz cuvettes until the maximum response fell within the detection limit of the instrument. Fluorescence was initially measured at an excitation wavelength λ_{ex} of 365 nm, although λ_{ex} was further optimized if needed. PF4SN homopolymers, and F8 and Ph copolymers were excited at 365 nm, while BT at 400 nm, and T at 415 nm.

Wide-angle X-ray scattering measurements (WAXS) were performed at the MINA diffractometer of the University of Groningen. This setup is equipped with a Cu rotating anode, emitting X-rays of a wavelength of 1.5413 Å (8 keV), and a Bruker Vantec 500 2D detector with a pixel size of 136 × 136 μm. The beam size measured 250 × 250 μm. A sample-to-detector distance of 80 mm was used for experiments in transmission mode. Samples were accommodated in metallic washers that were secured with Kapton tape, and depending on the scattering power of each sample, the acquisition time was varied from 5 to 15 min in order to have very low signal-to-noise ratios and collect high quality data. The obtained patterns were converted into 1D intensity profiles using the freeware program Fit2D (ESRF, Grenoble, France) and the pixel scale was converted into the q-scale ($q = 4\pi/\lambda \sin\theta$) using the known peak positions of a silver behenate standard. The background of an empty metallic washer secured with Kapton tape was subtracted after proper scaling for the sample absorption. To allow direct comparison of the different copolymers, scattering profiles were normalized with respect to their maximum baseline intensity.

2 Polymer synthesis

Entry	Method	Solvent	Base	[Base] (M)	Catalyst	PTC	T (°C)	t (h)	Yield (%)	M_n	X_n	Đ
1	A	X	Na_2CO_3	2.0	$Pd(PPh_3)_4$	N	100	24	38	4.48	6.9	1.87
2	A	X	Na_2CO_3	2.0	$Pd(PPh_3)_4$	Y	100	22	67	6.63	10.2	2.24
3	A	D/X	K_2CO_3	4.0	$Pd(PPh_3)_4$	N	90	24	42	5.14	7.9	2.94
4	A	D/X	K_2CO_3	4.0	$Pd(PPh_3)_4$	Y	90	24	83	7.64	11.7	2.28
5	A	D/X	K_2CO_3	4.0	$Pd(PPh_3)_4$	Y	90	48	83	7.27	11.1	2.27
6	B	DMF	K_3PO_4	N/A	$Pd(dppf)Cl_2$	Y	80	30	53	7.86	12.0	3.33

Table S1: Optimization of the synthesis of PF4SN-Ph via Suzuki polycondensation. Details about the methods (A and B) are described in the “Polymerizations” section. Solvents: *o*-xylene (X), 1,4-dioxane (D), 1/1 mixtures thereof (D/X), or *N,N*-dimethylformamide (DMF). PTC indicates the use of phase-transfer catalyst (Aliquat 336). Yields were determined gravimetrically after precipitation and drying of the product, and molecular weights M_n (kg mol^{-1}) and their distribution Đ were determined by GPC (PS standards). X_n is the number-average degree of polymerization, calculated from M_n using the mass of the repeating unit.

Entry	Polymer	Method	Solvent	Base	[Base] (M)	Catalyst	PTC	T (°C)	t (h)	Yield (%)	M_n	X_n	Đ
1	PF4SN-Ph	A	D/X	K_2CO_3	4.0	$Pd(PPh_3)_4$	Y	90	24	83	7.64	11.7	2.28
2	PF4SN-BT	A	D/X	K_2CO_3	4.0	$Pd(PPh_3)_4$	Y	90	24	82	7.83	11.0	2.21
3	PF4SN-F8	A	D/X	K_2CO_3	4.0	$Pd(PPh_3)_4$	Y	90	24	87	11.61	12.0	2.18
4	PF4SN-T	B	DMF	K_3PO_4	N/A	$Pd(dppf)Cl_2$	Y	80	24	75	6.30	9.6	2.28
5	PF4SN	A	D/X	K_2CO_3	4.0	$Pd(PPh_3)_4$	Y	90	24	74	9.60	16.6	2.03
6	PF4SN	C	DMF	K_3PO_4	N/A	$Pd(dppf)Cl_2$	Y	80	24	45	5.72	9.9	1.90

Table S2: Reaction conditions for the preparation of protected CPEs via Suzuki polycondensation. Details about the methods (A, B, and C) are described in the “Polymerizations” section. Solvents: 1/1 mixtures of 1,4-dioxane and *o*-xylene (D/X), or *N,N*-dimethylformamide (DMF). PTC indicates the use of phase-transfer catalyst (Aliquat 336). Yields were determined gravimetrically after precipitation and drying of the product, and molecular weights M_n (kg mol^{-1}) and their distribution Đ were determined by GPC (PS standards). X_n is the number-average degree of polymerization, calculated from M_n using the mass of the repeating unit.

3 Results and discussion

3.1 Monomers

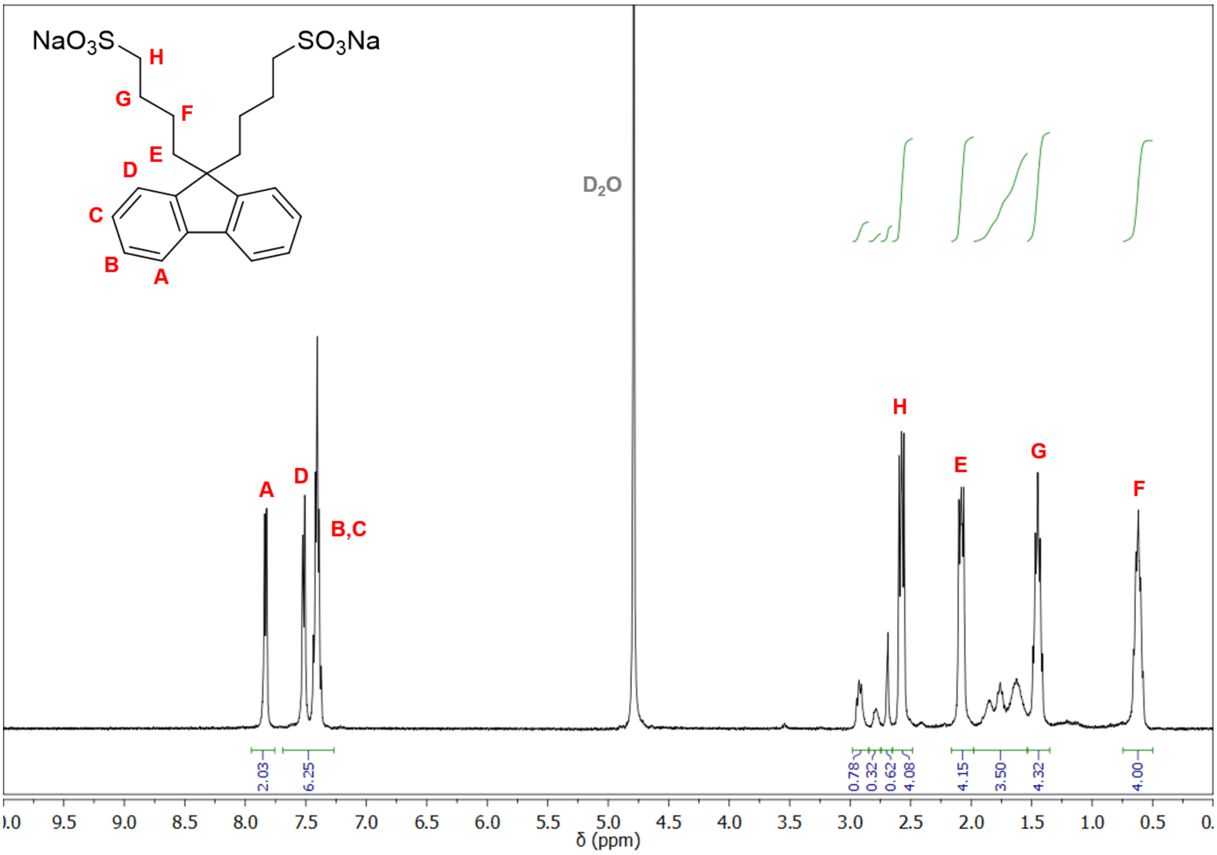

Figure S1: ^{1}H-NMR spectrum of crude sodium 4,4'-(9H-fluorene-9,9-diyl)bis(butane-1-sulfonate) (F4SO_3Na) recorded in D_2O. The product was used in the next step without further purification.

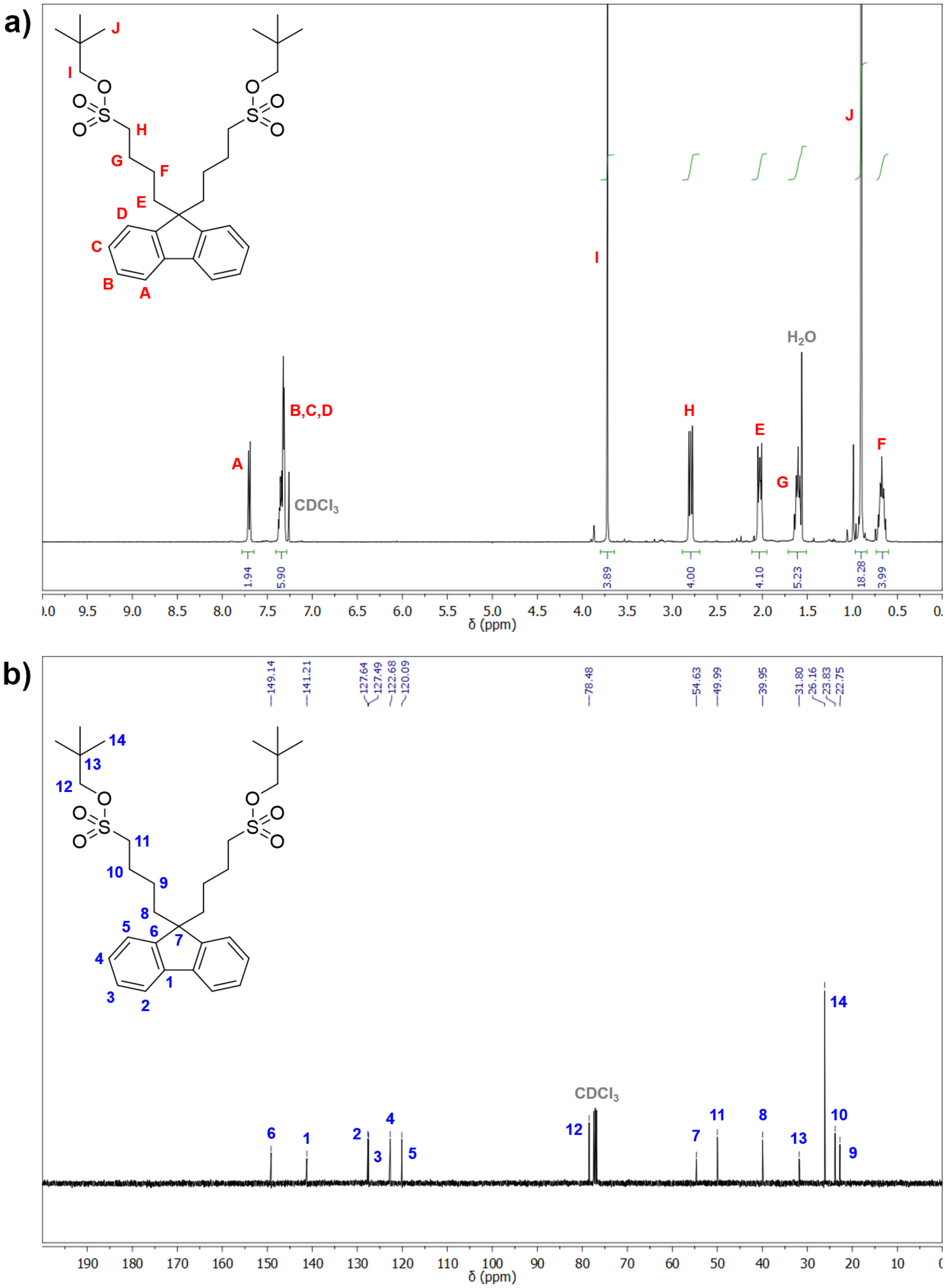

Figure S2: (a) ^{1}H-NMR and (b) ^{13}C-NMR spectra ($CDCl_3$) of purified dineopentyl 4,4'-(9H-fluorene-9,9-diyl)bis(butane-1-sulfonate) (F4SN).

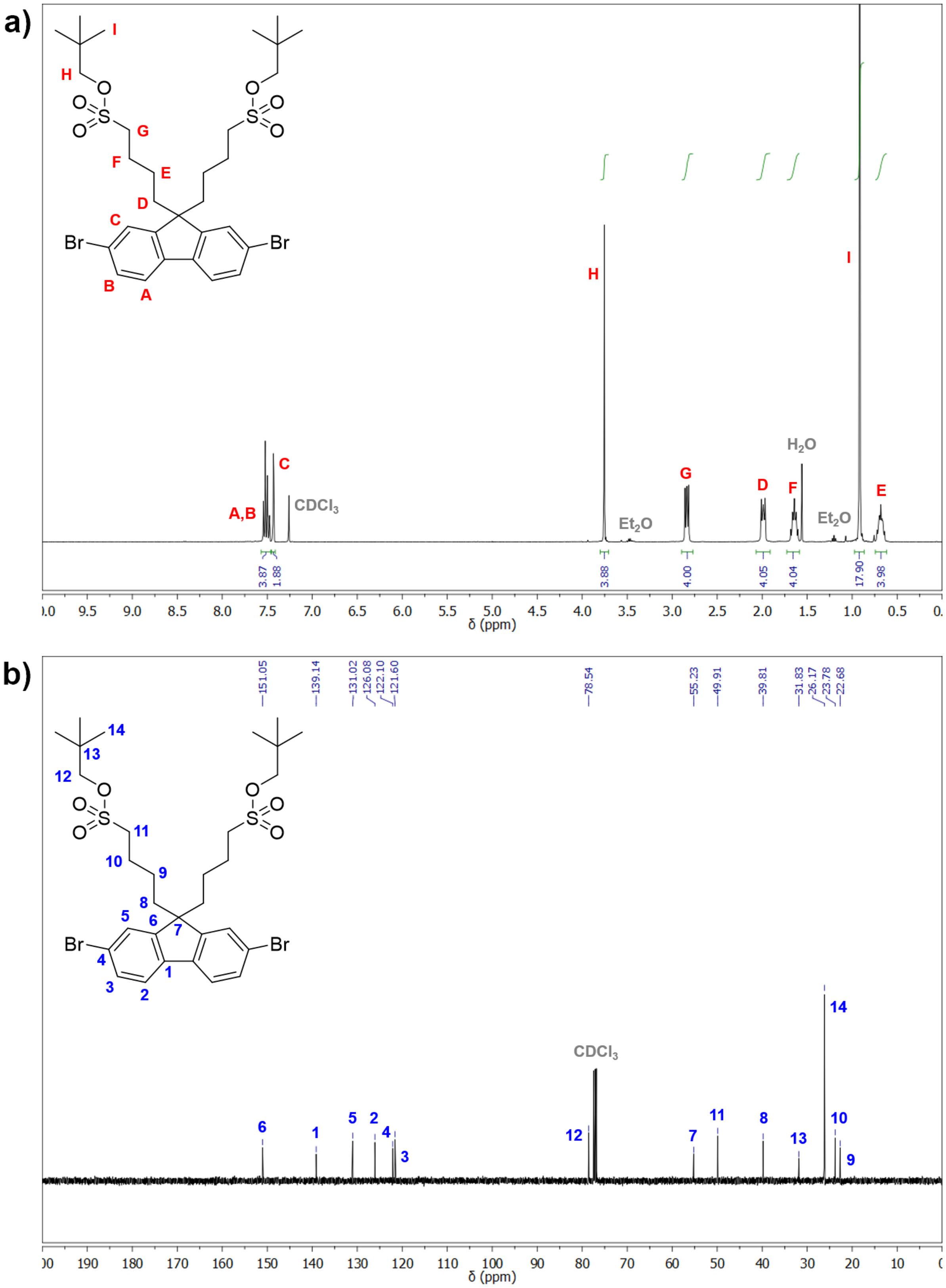

Figure S3: (a) ^{1}H-NMR and (b) ^{13}C-NMR spectra ($CDCl_3$) of purified dineopentyl 4,4'-(2,7-dibromo-9H-fluorene-9,9-diyl)bis(butane-1-sulfonate) (F4SN-Br_2).

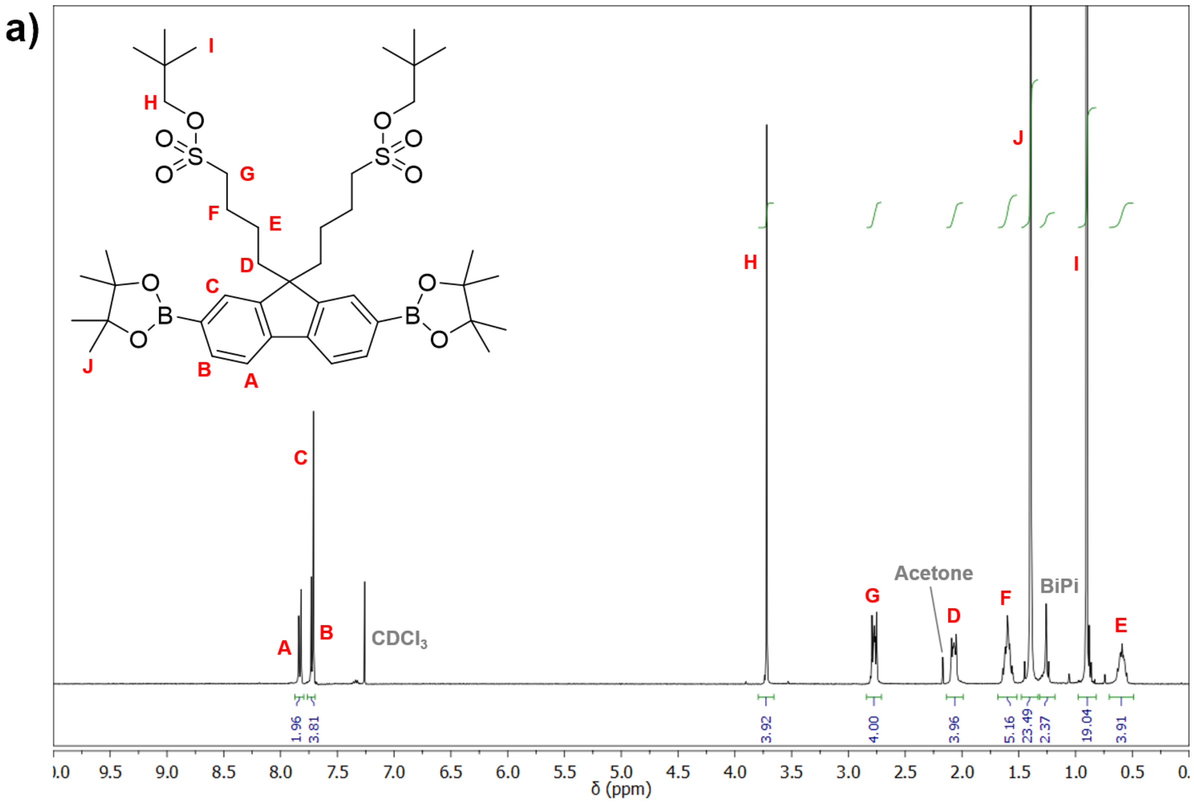

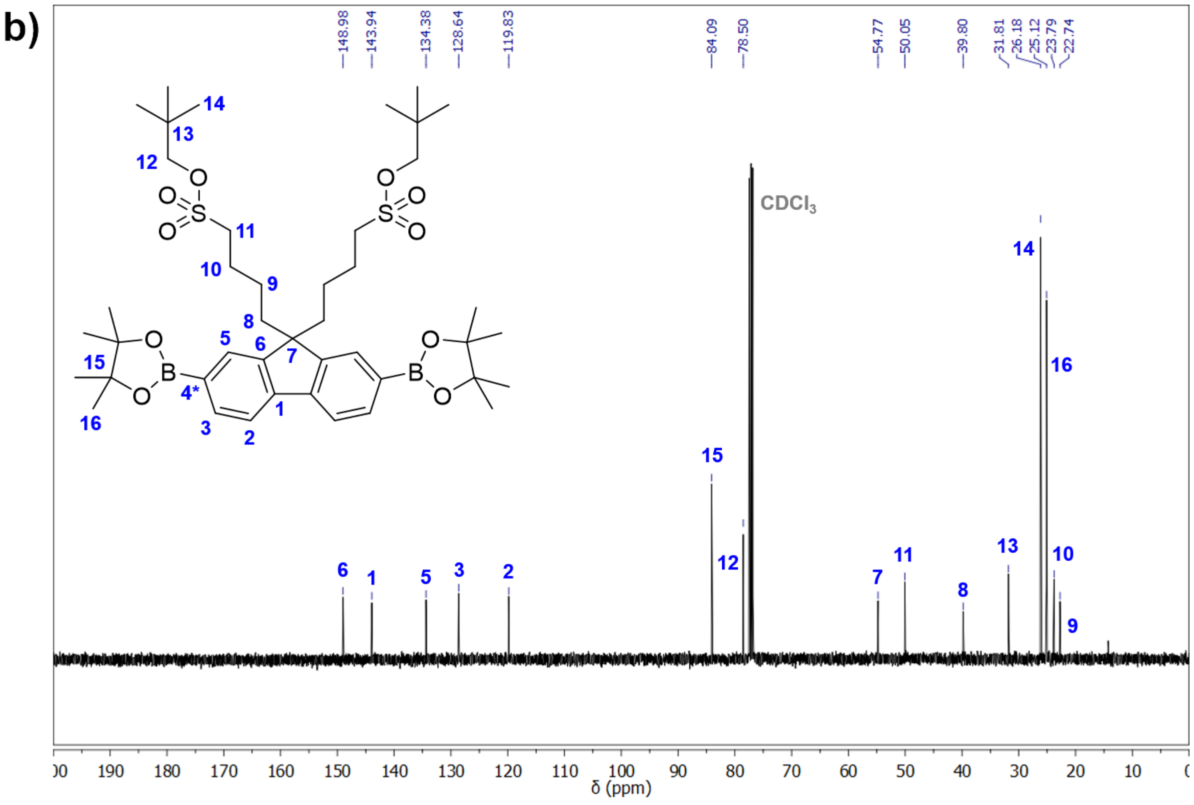

Figure S4: (a) ^{1}H-NMR and (b) ^{13}C-NMR spectra ($CDCl_3$) of purified dineopentyl 4,4'-(2,7-bis(4,4,5,5-tetramethyl-1,3,2-dioxaborolan-2-yl)-9H-fluorene-9,9-diyl) bis(butane-1-sulfonate) (F4SN-$Bpin_2$). Carbon [4*] is not visible due to ^{13}C-B coupling.

3.2 Polymerizations

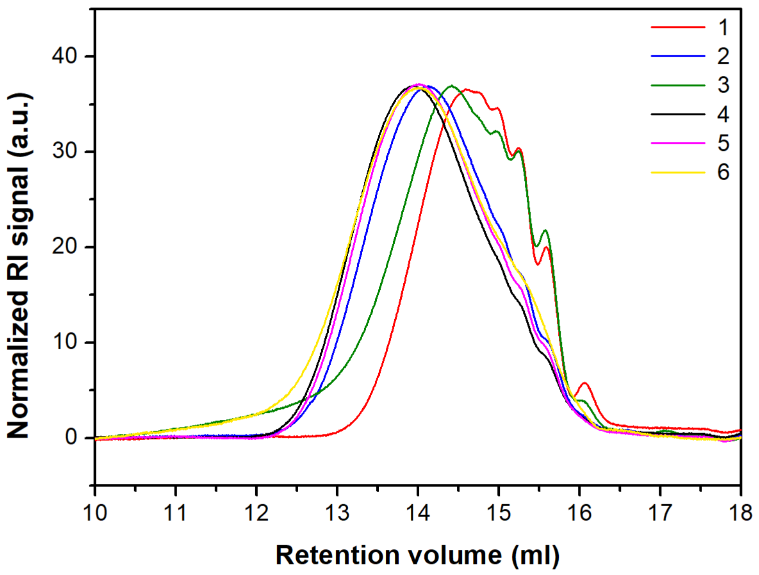

Figure S5: GPC chromatograms of PF4SN-Ph copolymers synthesized using various strategies and reaction conditions. Numbers indicate the experiments listed in **Table S1**.

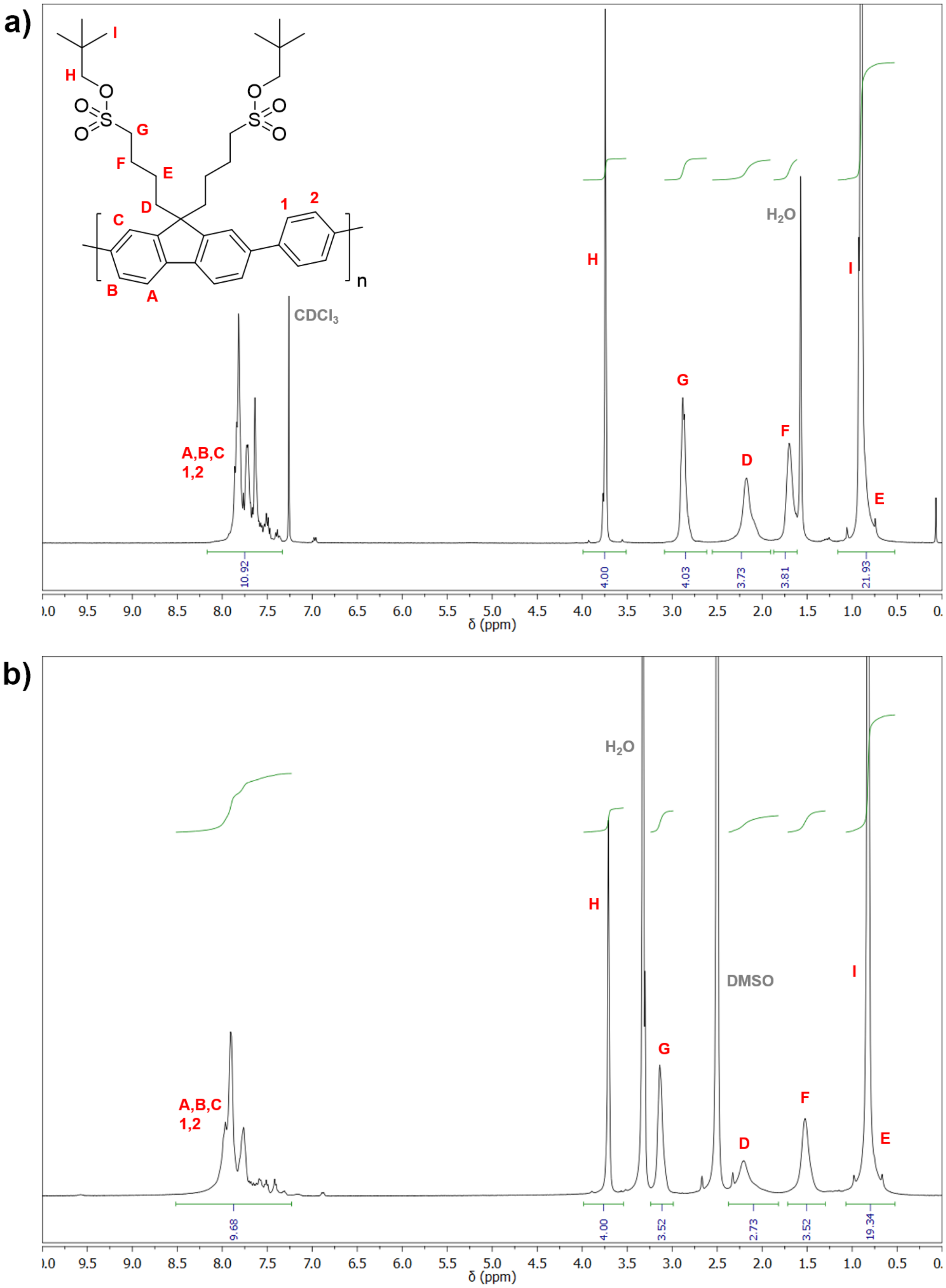

Figure S6: ^{1}H-NMR spectra of purified PF4SN-Ph recorded in (a) $CDCl_3$ and (b) DMSO-d_6.

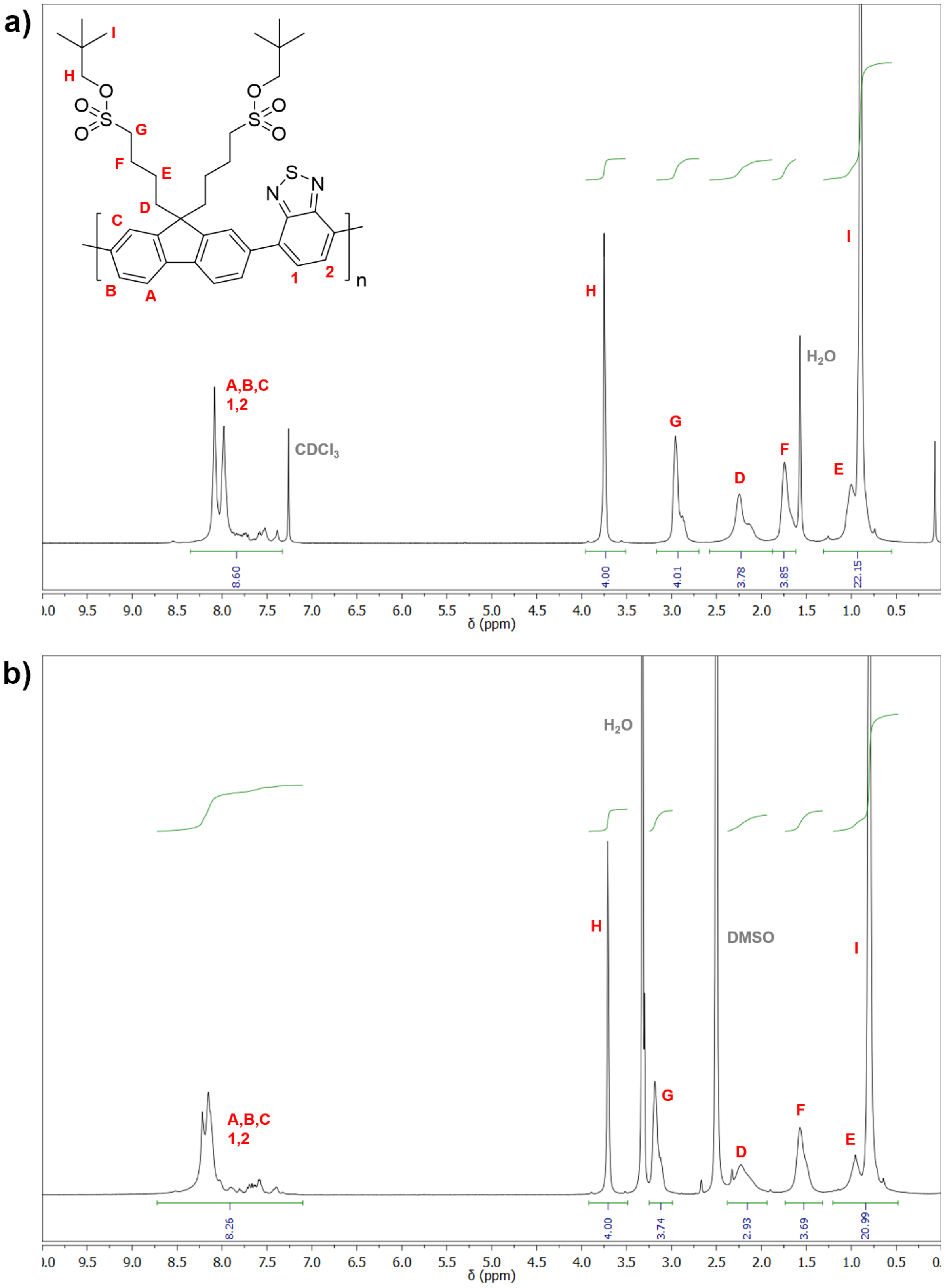

Figure S7: ^{1}H-NMR spectra of purified PF4SN-BT recorded in (a) $CDCl_3$ and (b) DMSO-d_6.

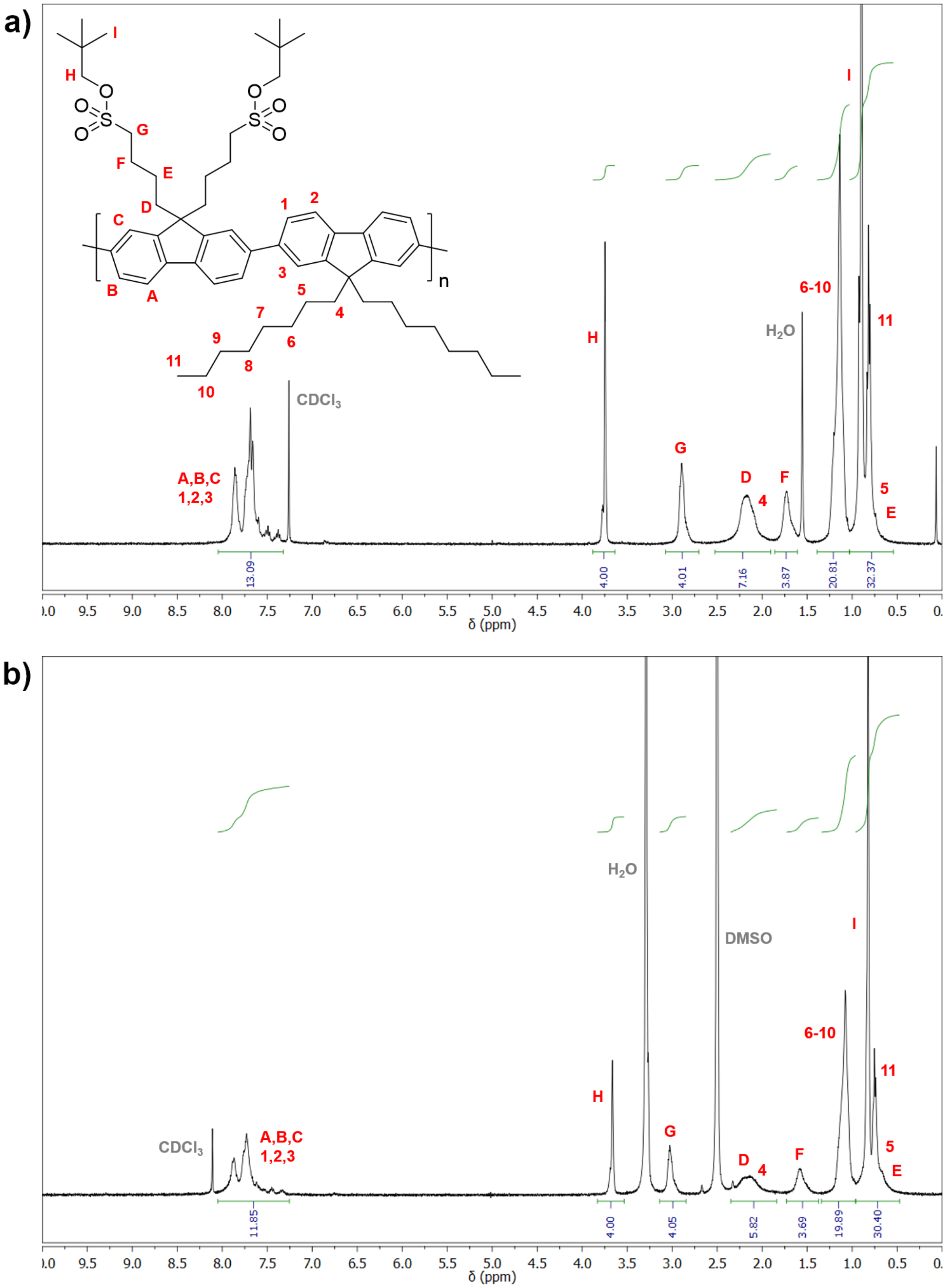

Figure S8: ^{1}H-NMR spectra of purified PF4SN-F8 recorded in (a) $CDCl_3$ and (b) a 1/1 mixture of $CDCl_3$ and DMSO-d_6. PF4SN-F8 is not soluble in DMSO at room temperature.

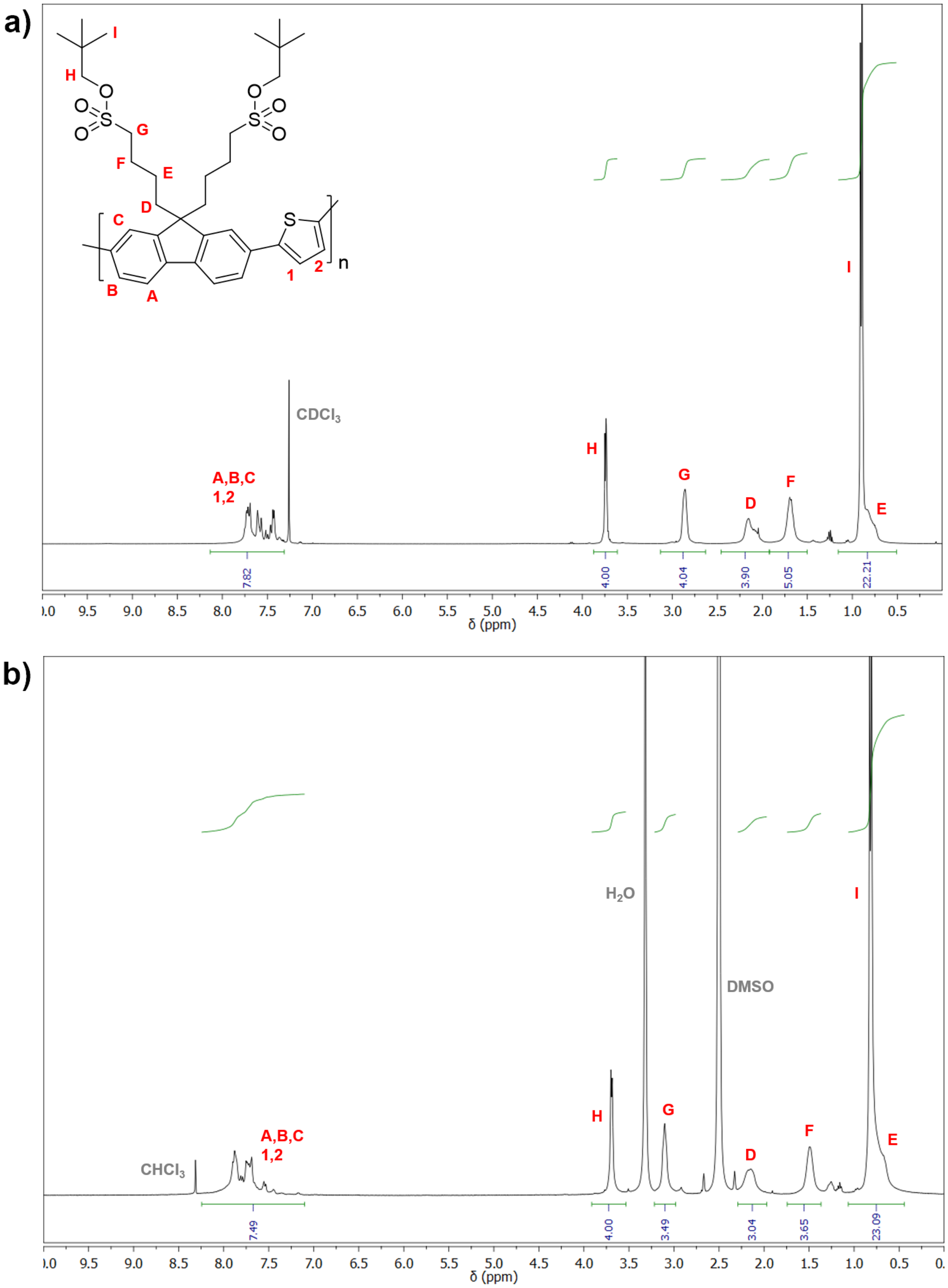

Figure S9: ^{1}H-NMR spectra of purified PF4SN-T recorded in (a) $CDCl_3$ and (b) DMSO-d_6.

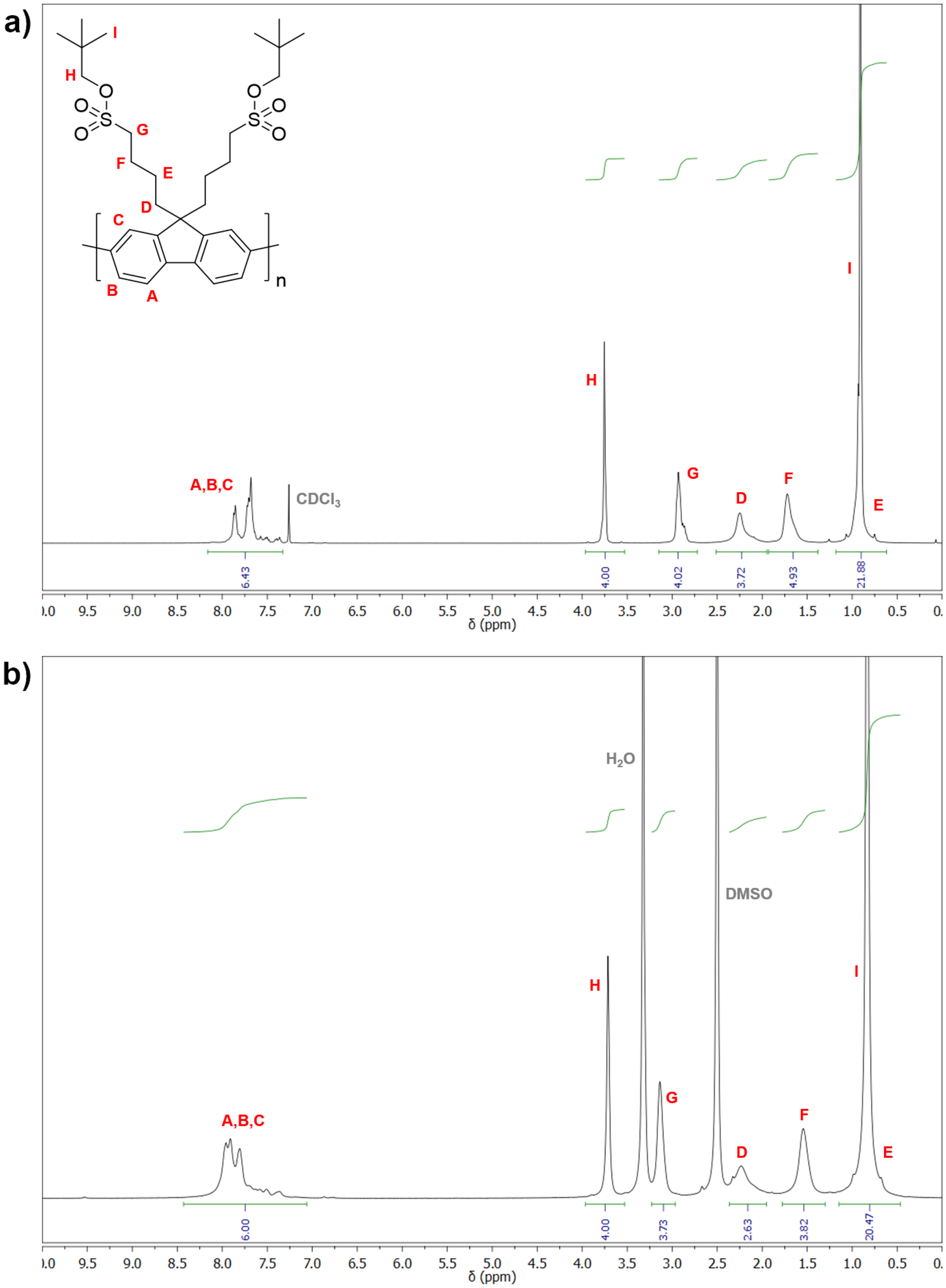

Figure S10: ^{1}H-NMR spectra of purified PF4SN recorded in (a) $CDCl_3$ and (b) DMSO-d_6. This PF4SN sample was synthesized via method A (**Table S2, entry 5**).

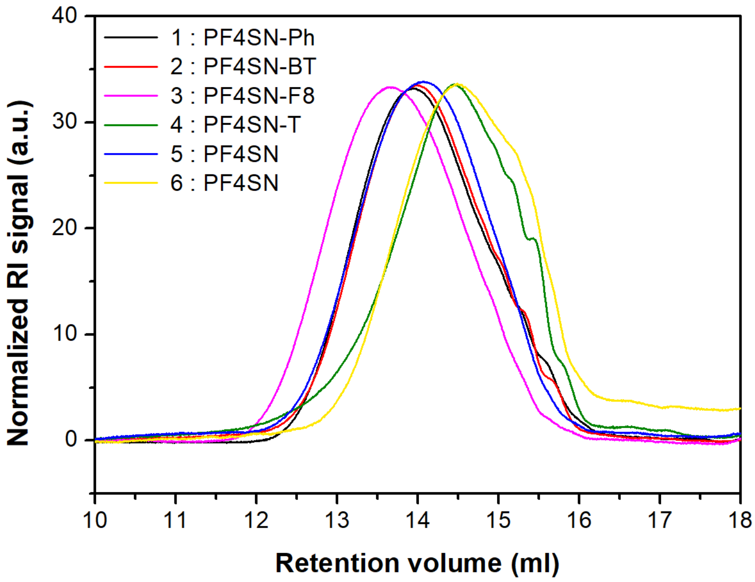

Figure S11: GPC chromatograms of PF4SN copolymers. Numbers indicate the experiment numbers outlined in **Table S2**. Note that comparison of the elution volumes should be done with some caution, as the different copolymers were not analyzed in the same run.

3.3 Polymer deprotection

a)

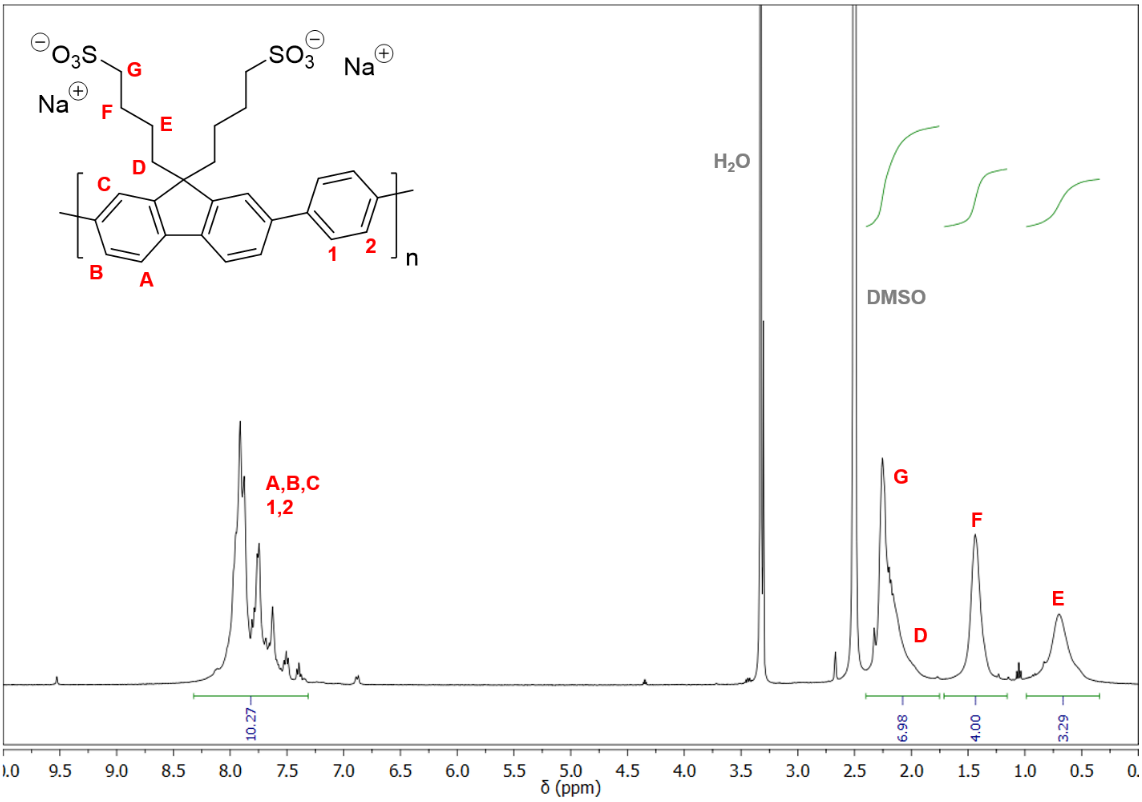

b)

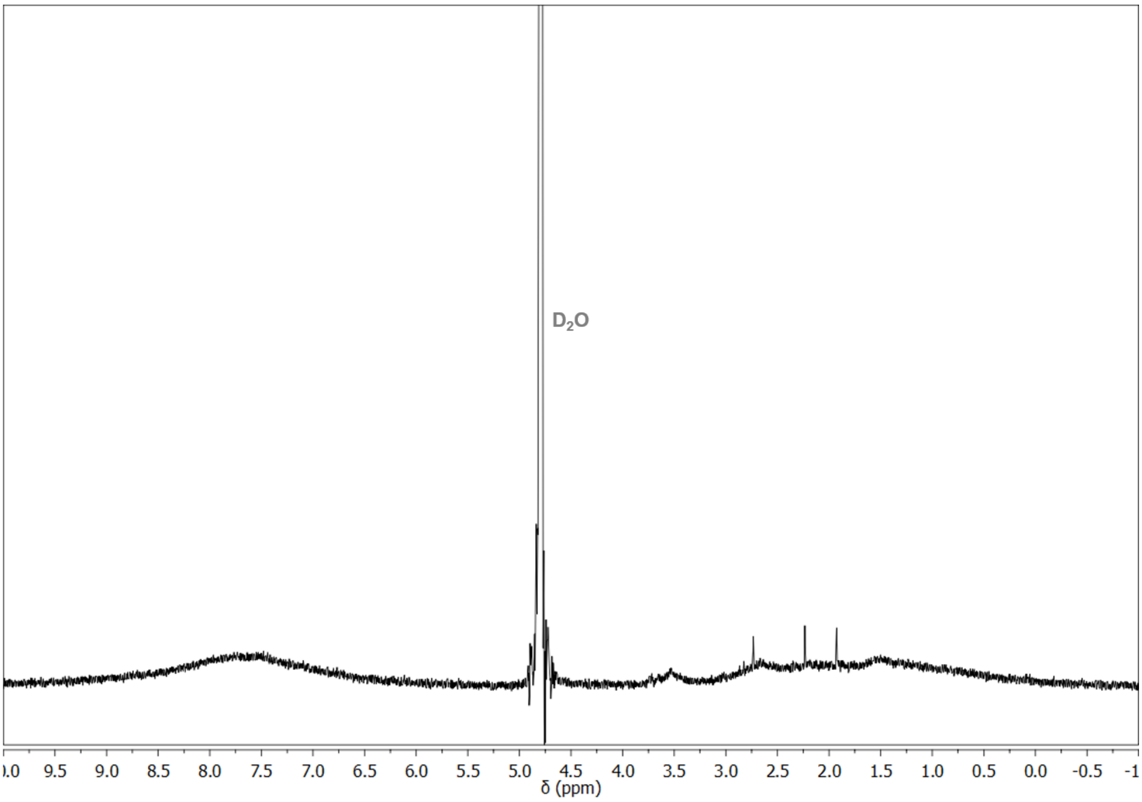

Figure S12: ^{1}H-NMR spectra of PF4SO$_3$Na-Ph recorded in (a) DMSO-d$_6$ and (b) D$_2$O. Aggregation of the CPE in water results in broad, non-informative signals.

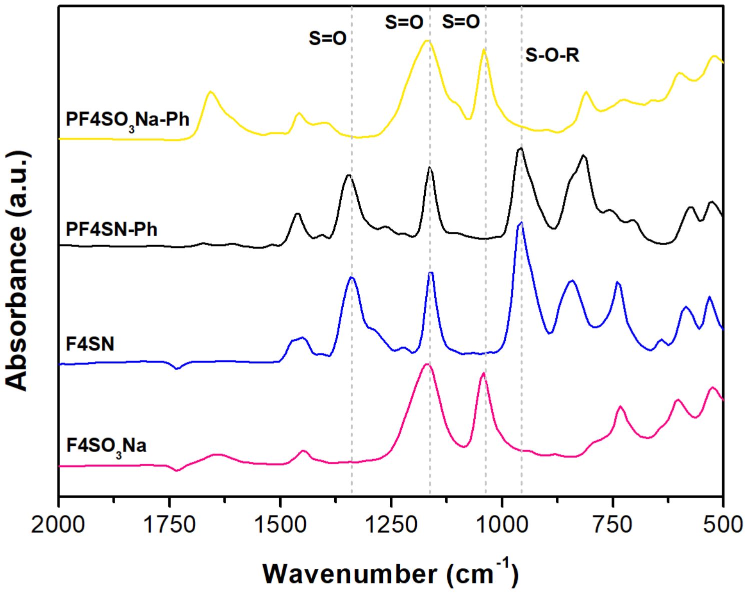

Figure S13: FTIR spectra of $F4SO_3Na$, F4SN, PF4SN-Ph, and $PF4SO_3Na$-Ph. Protection and deprotection of the monomer and polymer can be followed qualitatively by monitoring the sulfonate (1040 and 1164 cm^{-1}) and sulfonic ester (958 and 1340 cm^{-1}) stretching modes.

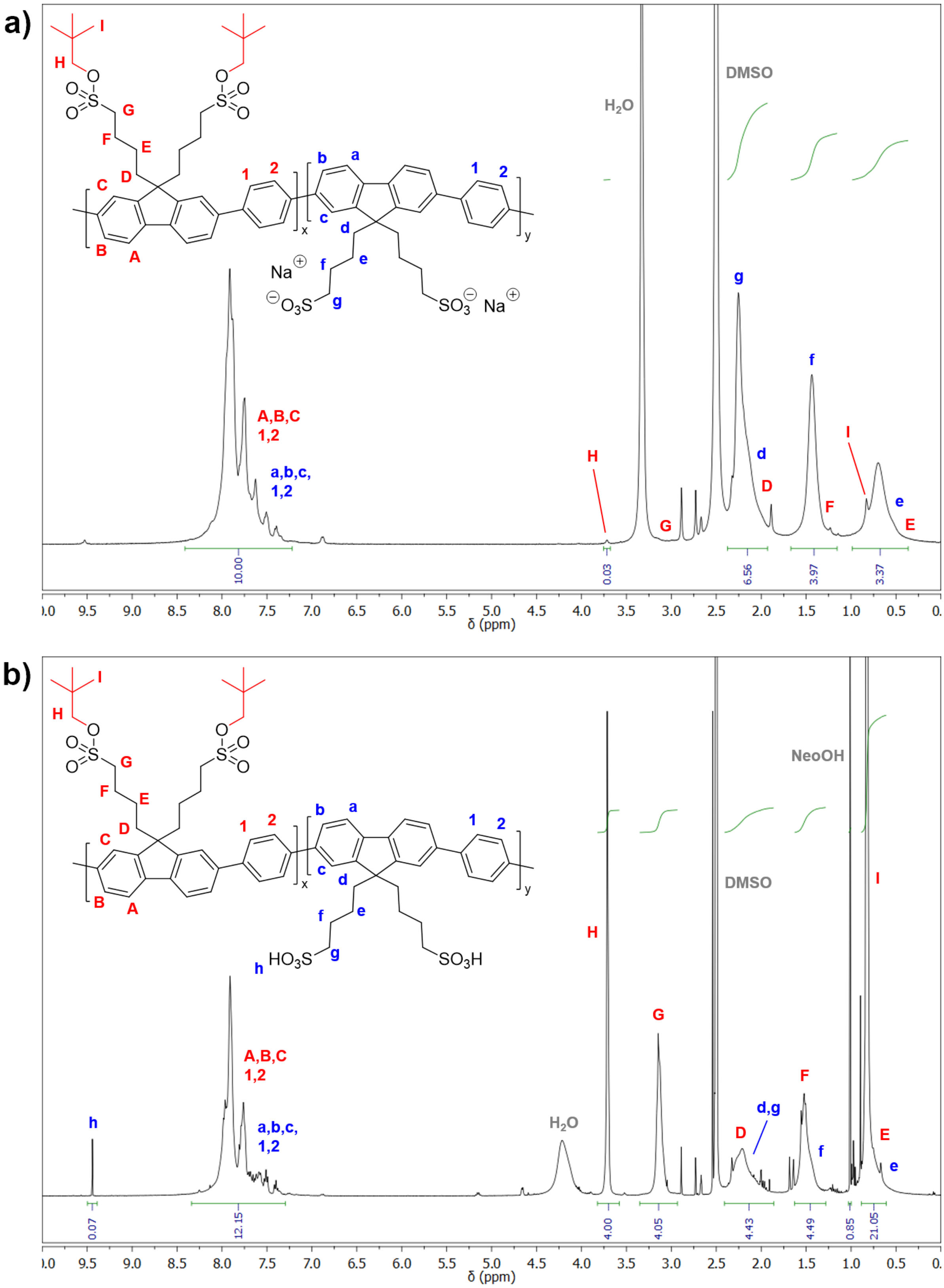

Figure S14: (a) Treatment of PF4SN-Ph with NaN_3 at 110 °C for 72 h resulted in > 99% removal of the neopentyl protecting groups (ratio aromatic signals and protons [H]). (b) Control experiment performed in DMSO-d_6. Heating at 130 °C for 23 h in the absence of NaN_3 resulted in only 10 – 20% deprotection, depending on the calculation method. Comparison protons [H] and [F] and [f]: 11%. Aromatic protons and [H]: 18%.

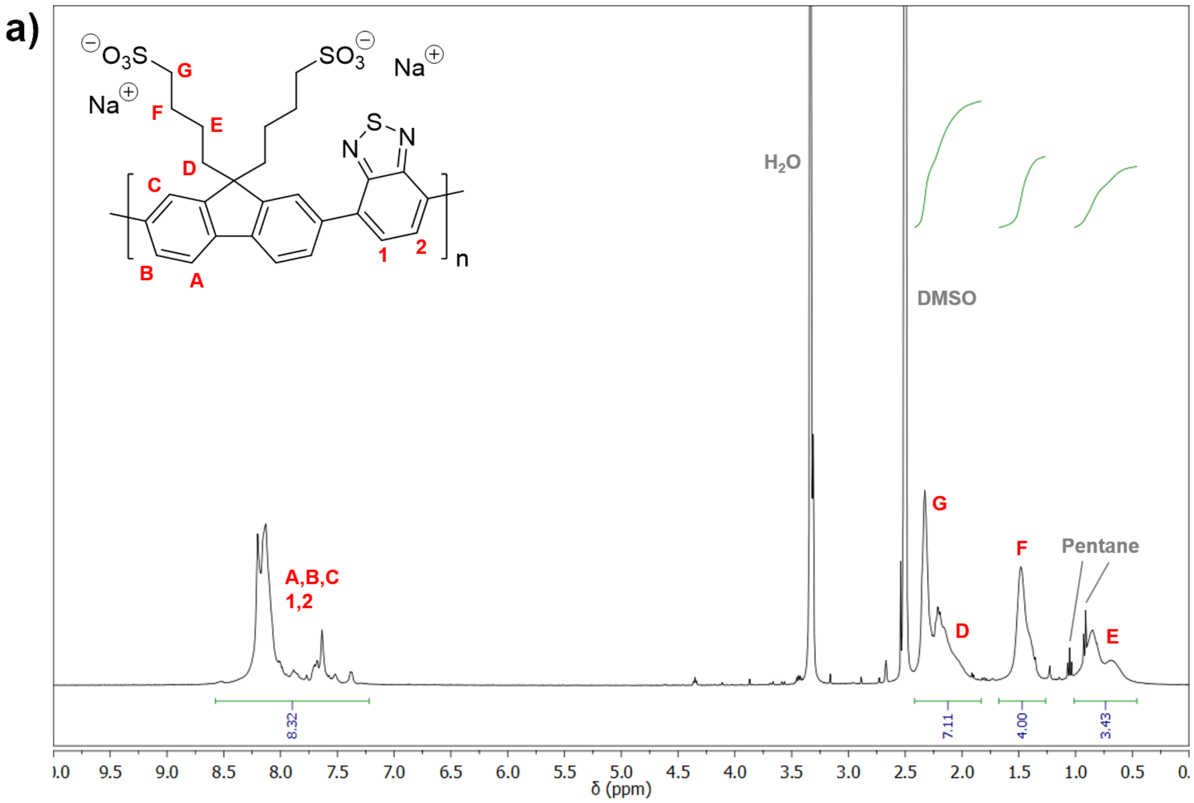

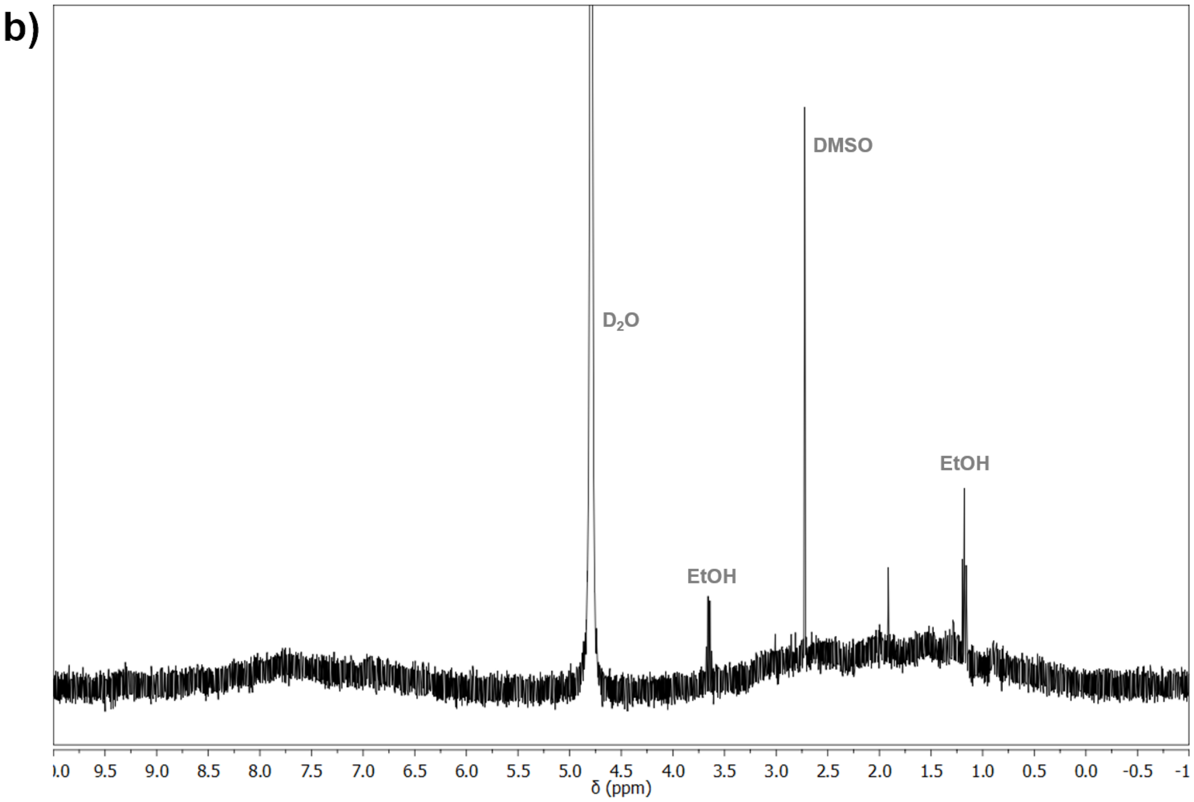

Figure S15: ^{1}H-NMR spectra of PF4SO$_3$Na-BT recorded in (a) DMSO-d$_6$ and (b) D$_2$O. Aggregation of the CPE in water results in broad, non-informative peaks. Only signals originating from traces of residual solvent can be identified.

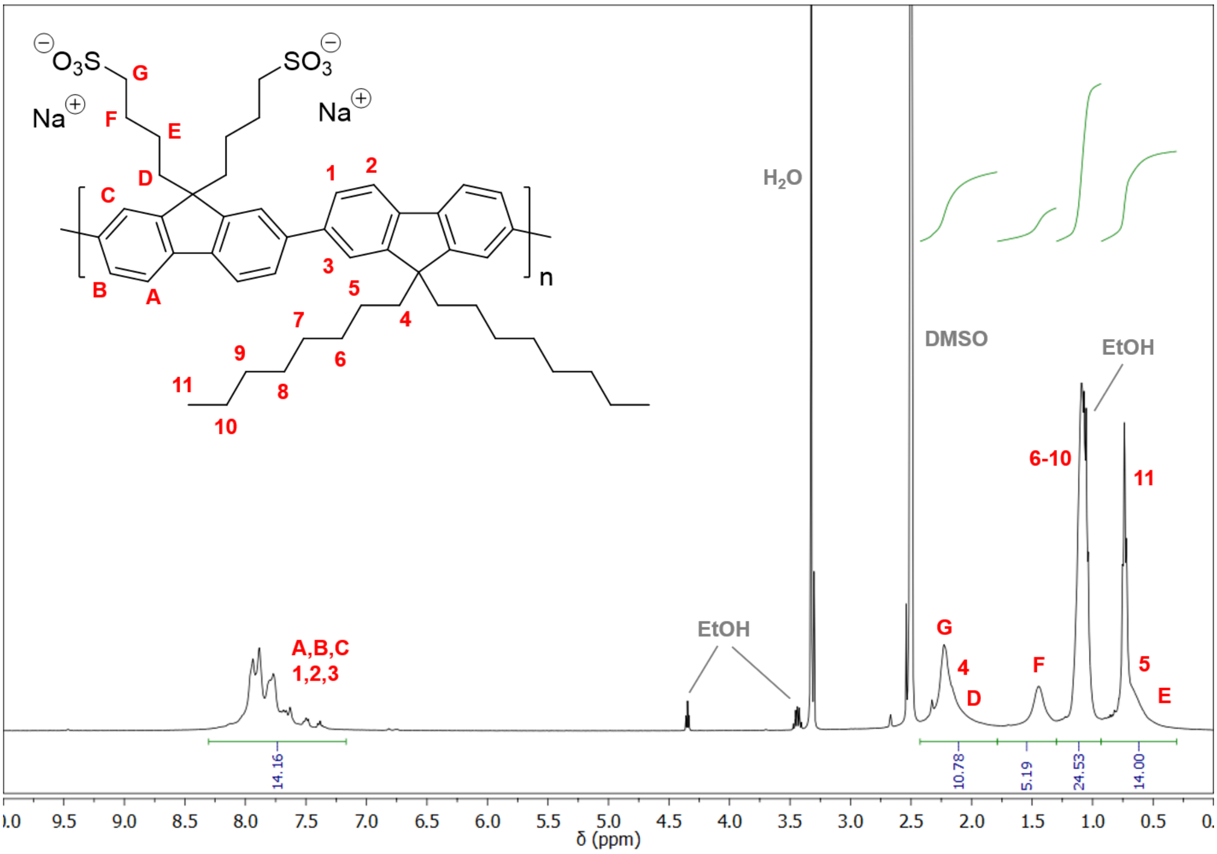

Figure S16: ^{1}H-NMR spectrum of PF4SO$_3$Na-F8 recorded in DMSO-d$_6$. PF4SO$_3$Na-F8 is not soluble in D$_2$O.

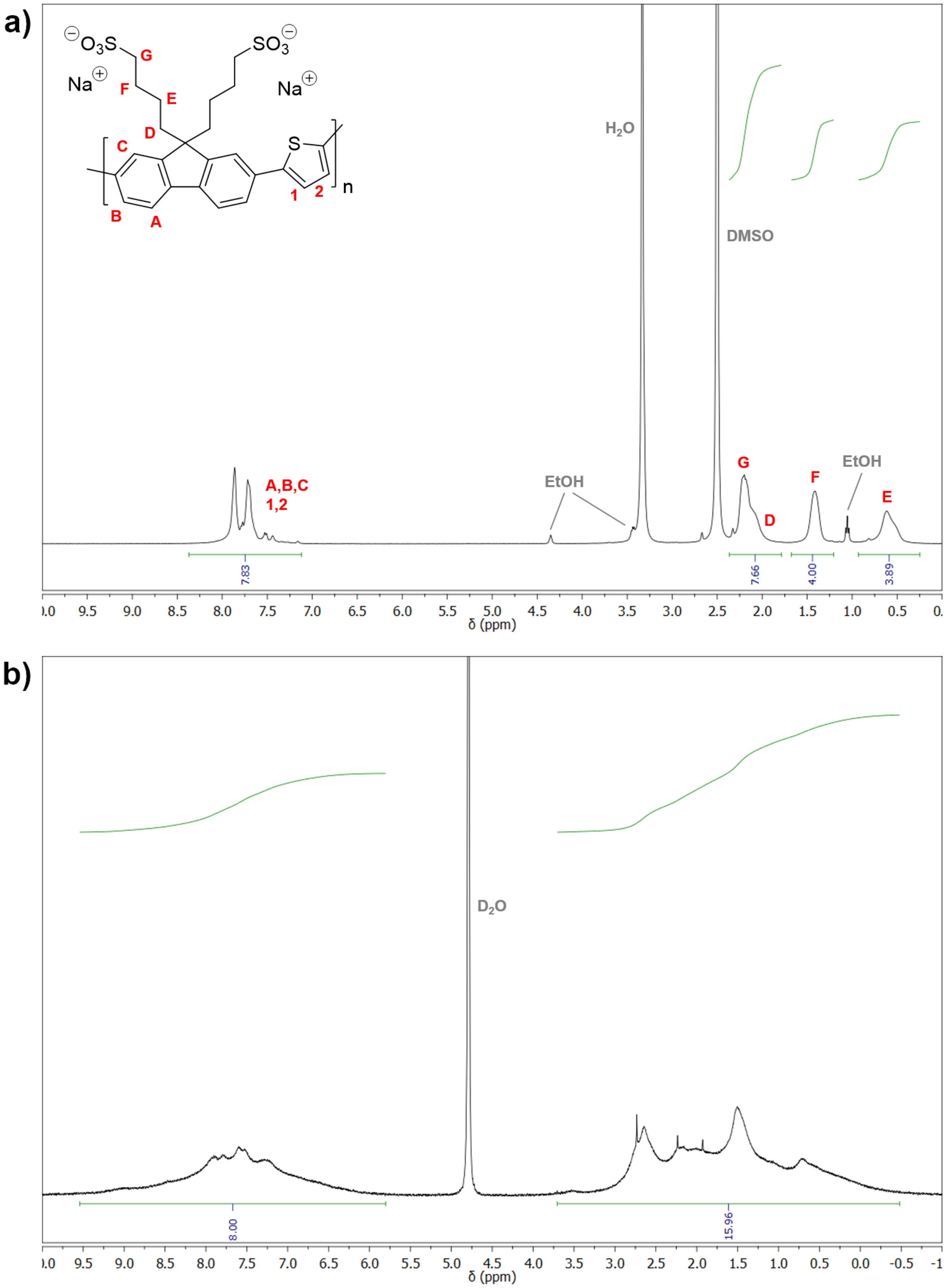

Figure S17: ^{1}H-NMR spectra of PF4SO$_3$Na-T recorded in (a) DMSO-d$_6$ and (b) D$_2$O. Aggregation of the CPE in water results in broad, non-informative signals.

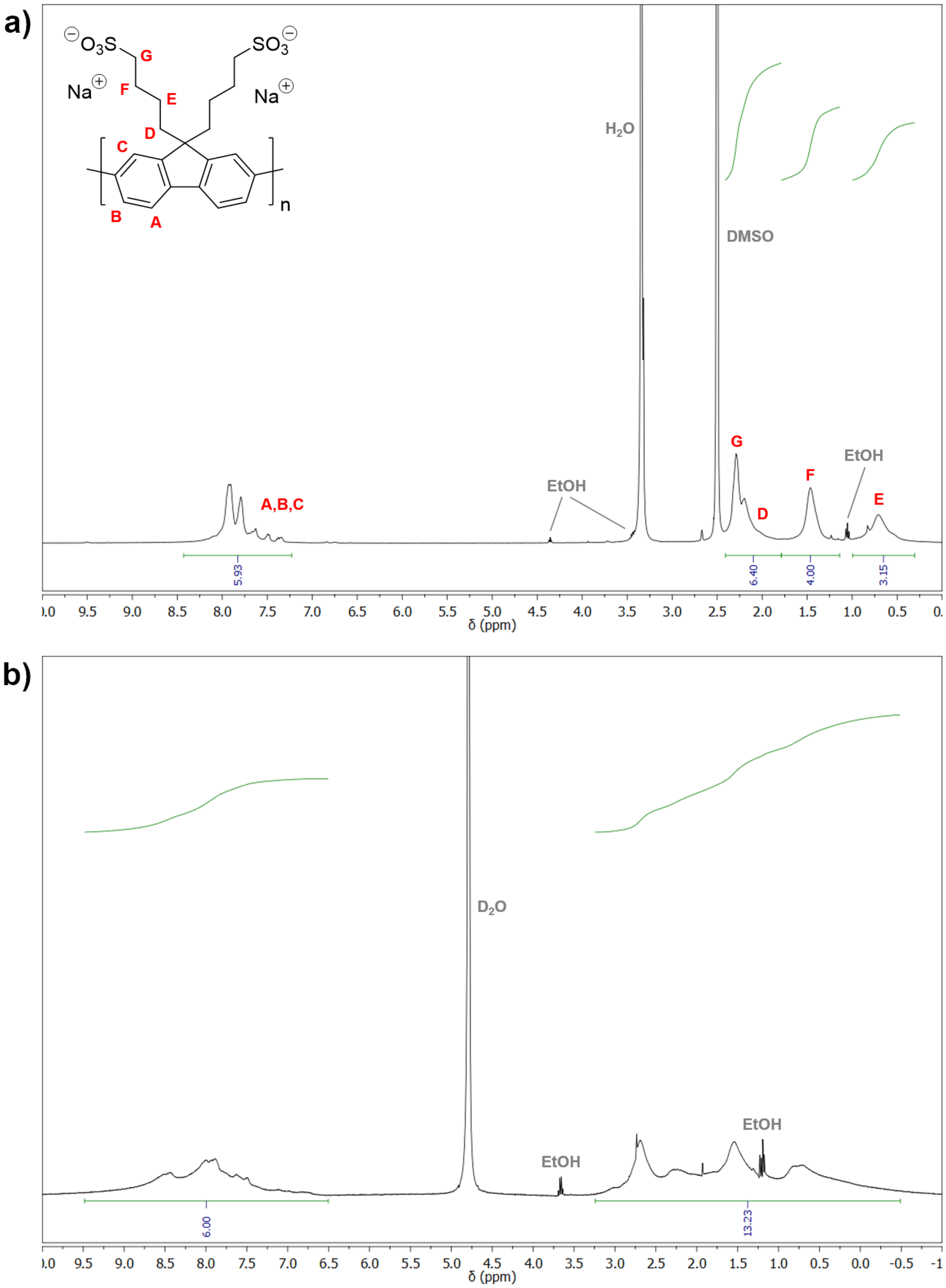

Figure S18: ^{1}H-NMR spectra of PF4SO$_3$Na recorded in (a) DMSO-d$_6$ and (b) D$_2$O. Aggregation of the CPE in water results in broad, non-informative peaks. Only signals originating from traces of residual solvent can be identified.

3.4 Photophysical properties

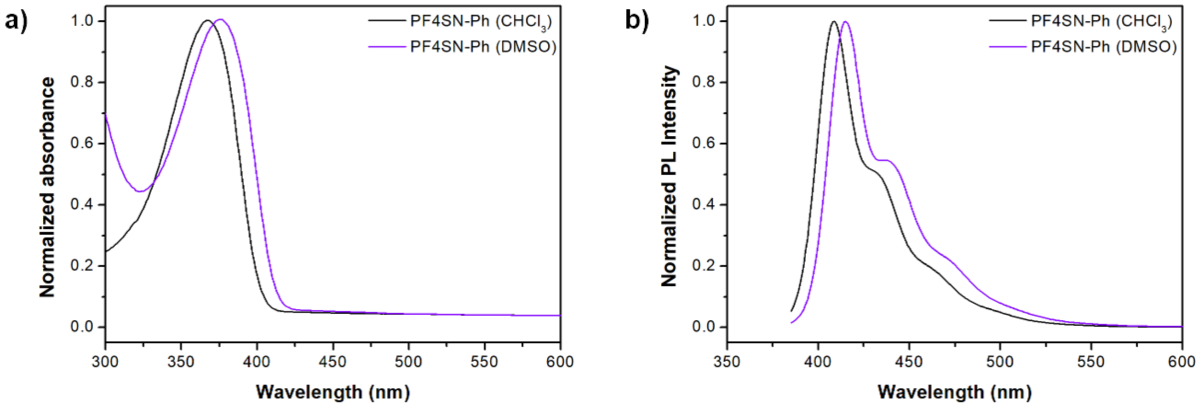

Figure S19: Effect of the solvent on the absorption and emission of PF4SN-Ph. Chloroform vs DMSO: normalized (a) UV-Vis and (b) PL spectra (λ_{ex} = 365 nm).

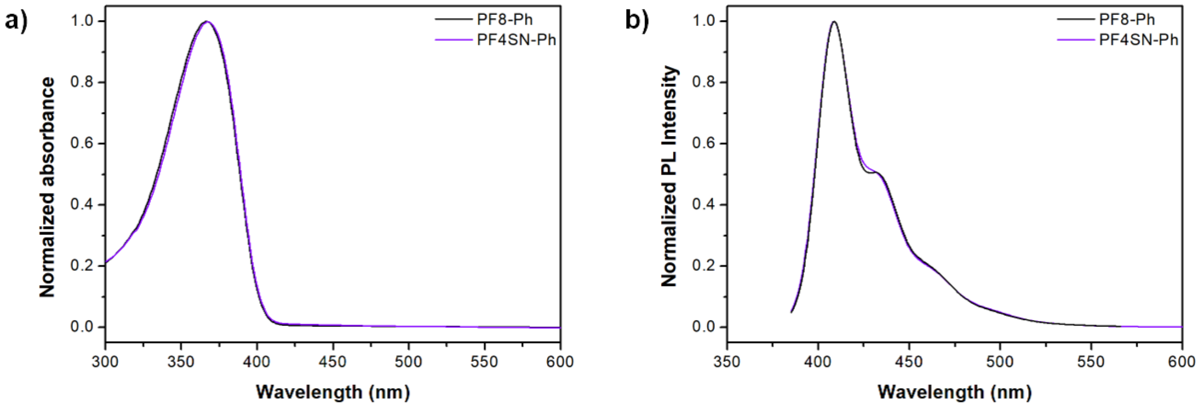

Figure S20: Comparison of the photophysical properties of PF4SN-Ph and PF8-Ph, recorded in chloroform. Normalized (a) UV-Vis and (b) PL spectra (λ_{ex} = 365 nm).

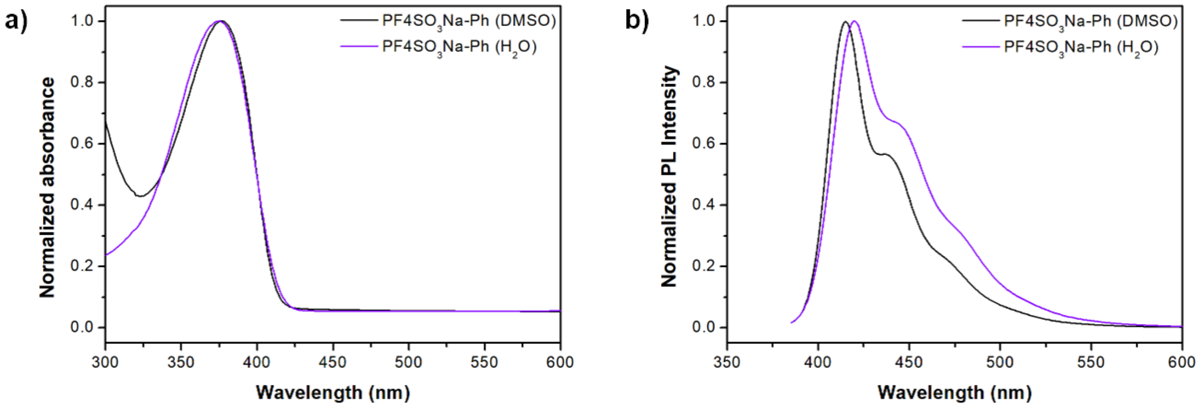

Figure S21: Effect of the solvent on the absorption and emission of PF4SO_3Na-Ph. Water vs DMSO: normalized (a) UV-Vis and (b) PL spectra (λ_{ex} = 365 nm).

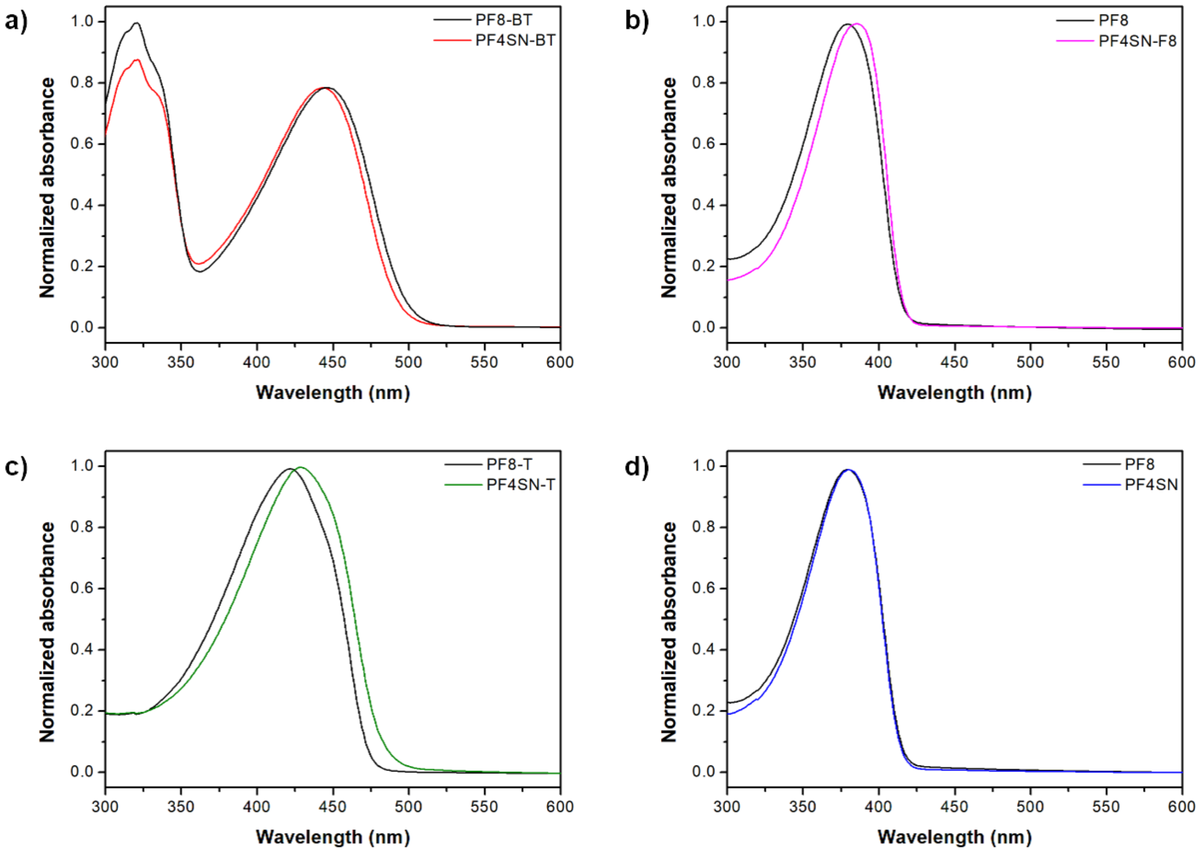

Figure S22: Comparison of the photophysical properties of PF4SN and PF8 conjugated donor-acceptor copolymers by UV-Vis spectroscopy, recorded in chloroform. (a) PF4SN-BT vs PF8-BT, (b) PF4SN-F8 vs PF8, (c) PF4SN-T vs PF8-T, and (d) PF4SN vs PF8.

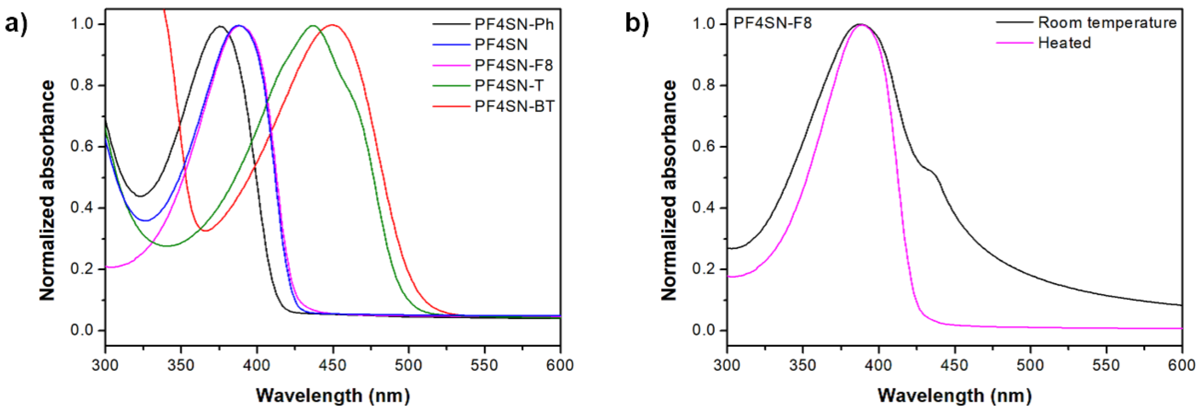

Figure S23: (a) Normalized UV-Vis spectra of all PF4SN-based donor-acceptor copolymers measured in DMSO. (b) UV-Vis absorption spectra of PF4SN-F8 in DMSO recorded at room temperature (aggregated/cloudy), and at higher temperature (dissolved/clear).

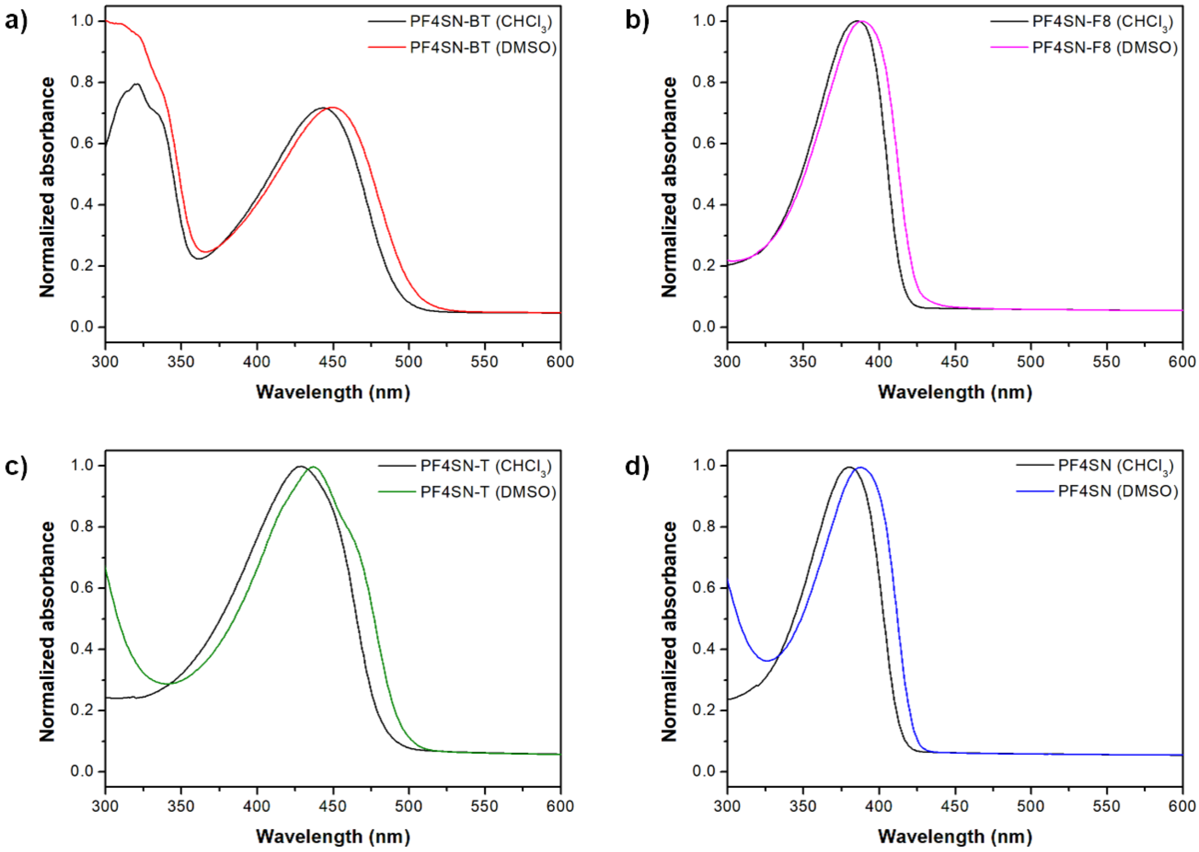

Figure S24: Effect of the solvent (chloroform vs DMSO) on the photophysical properties of PF4SN-based donor-acceptor copolymers. Normalized UV-Vis spectra of (a) PF4SN-BT, (b) PF4SN-F8, (c) PF4SN-T, and (d) PF4SN.

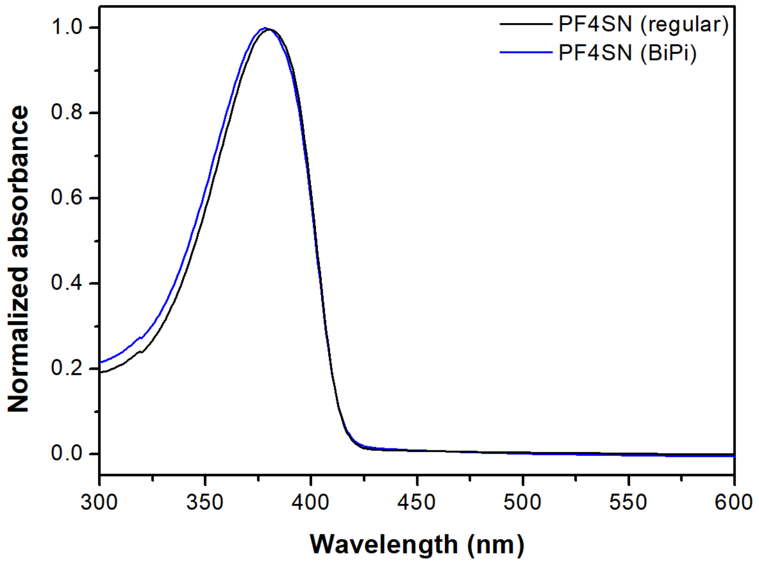

Figure S25: Normalized UV-Vis spectra of PF4SN homopolymer synthesized via the regular Suzuki polycondensation route (method A) and the BiPi route (method C). Spectra were recorded in chloroform.

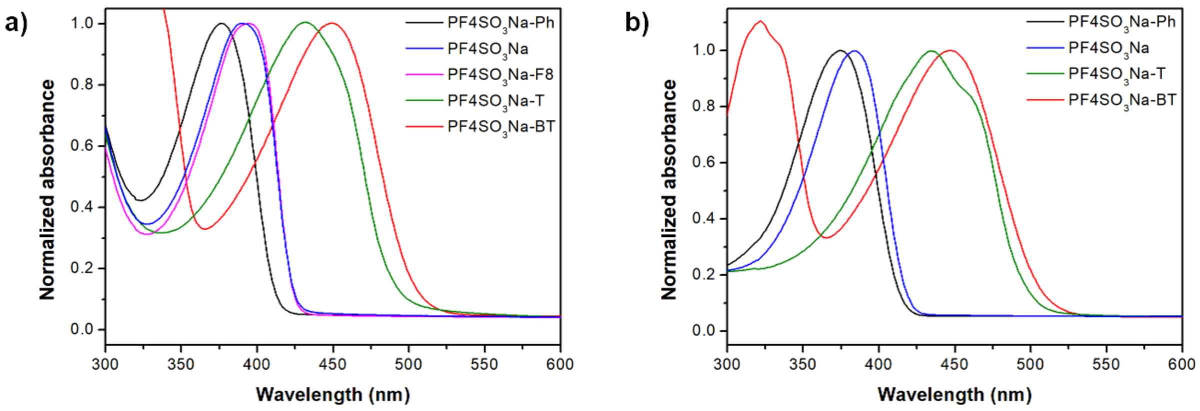

Figure S26: Normalized UV-Vis absorption spectra of all $PF4SO_3Na$-based donor-acceptor copolymers measured in (a) DMSO and (b) water. $PF4SO_3Na$-F8 is omitted in (b), since it is not soluble in water.

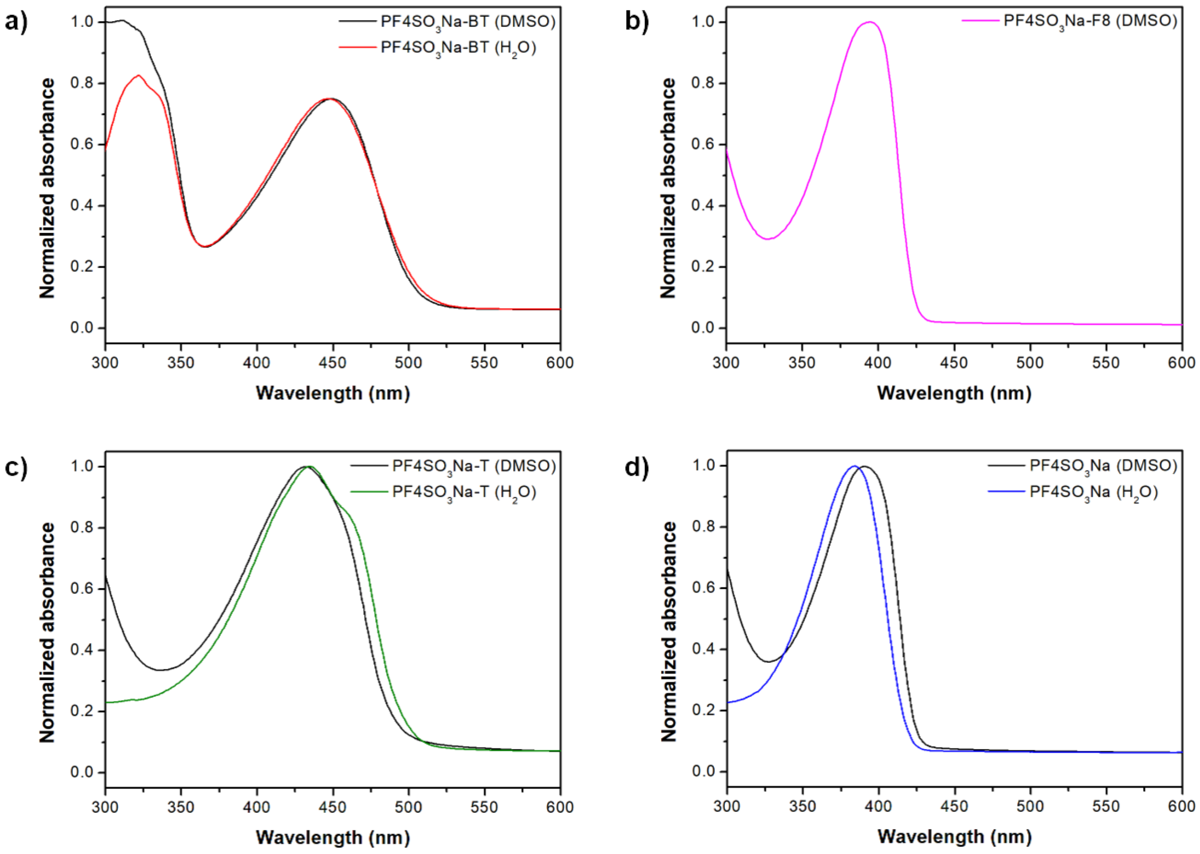

Figure S27: Effect of the solvent (DMSO vs water) on the photophysical properties of $F4SO_3Na$-containing CPEs. Normalized UV-Vis spectra of (a) $PF4SO_3Na$-BT, (b) $PF4SO_3Na$-F8 (water-insoluble), (c) $PF4SO_3Na$-T, and (d) $PF4SO_3Na$.

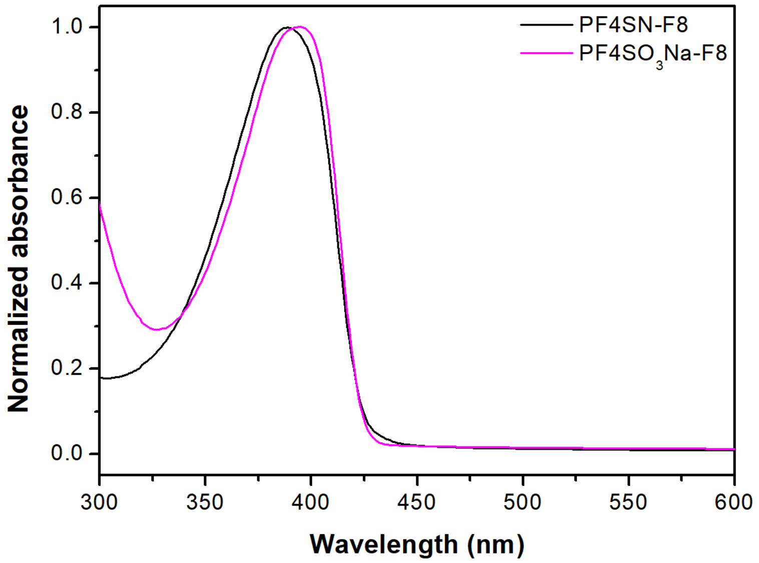

Figure S28: Normalized UV-Vis absorption spectra of PF4SN-F8 and PF4SO_3Na-F8, recorded in DMSO.

3.5 Thermal properties

Using TGA (**Figure S29**), the thermal removal of the neopentyl groups can be observed between 210 and 240 °C. The weight loss of this first degradation event, indicated by the red vertical arrow, matches the theoretical values remarkably well (**Table S3**), being the lowest for PF4SN-F8 and the highest for PF4SN. Rapid deprotection occurs on heating, since this process proceeds via an acid-catalyzed mechanism through the in-situ formed sulfonic acid moiety.[13] Interestingly, deprotection appears to be delayed for larger comonomers like BT (blue horizontal arrow) and is even more pronounced for F8 ($\Delta T \approx 25$ °C vs PF4SN-T). It is envisaged that larger neighbors cause an increased distance between the F4SN units, thereby protecting the sulfonate esters from the local acidic environment, which consequently slows down thermal deprotection.

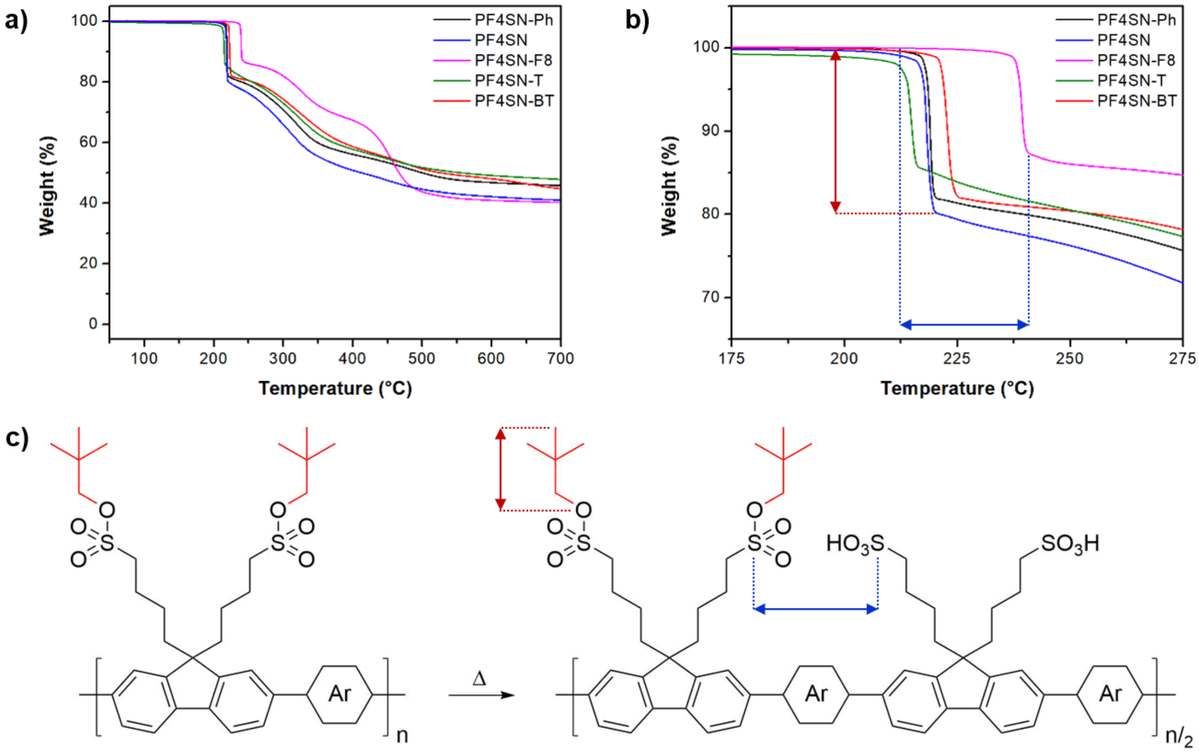

Figure S29: (a) Full and (b) zoomed-in degradation profiles of the protected CPEs obtained by TGA. (c) Thermal deprotection proceeds via an acid-catalyzed mechanism. The identity of the comonomer has an effect on both the relative weight loss upon thermal deprotection (red arrow), and the onset of thermal deprotection (blue arrow).

DSC was used to assess the glass transition temperatures (T_g) of the copolymers: they range from 90 °C for the most flexible PF4SN-F8 copolymer, up to 122 °C for the most rigid PF4SN-Ph copolymer (**Table S3** and **Figure S30**). These values are comparable to alkylated polyfluorenes, although one should be careful with drawing any conclusions from this data, as the T_g is also molecular weight-dependent.[14] Interestingly, a T_g could not be detected for PF4SN-T, which may be caused by either its weak response[15] or the transition being located above the maximum temperature that could be safely reached while avoiding thermal deprotection. The latter is not unthinkable, since the T_g of PF8-T is also significantly higher than that of other PF8 copolymers.[16]

Entry	Polymer	T_g (°C)	T_{onset} (°C)	T_{peak} (°C)	Wt (%)	Theory (%)
1	PF4SN-Ph	122	218	219	20.3	21.5
2	PF4SN-BT	114	221	222	19.2	19.7
3	PF4SN-F8	90	238	239	14.6	14.5
4	PF4SN-T	n.d.	214	215	19.5	21.3
5	PF4SN	101	217	218	22.3	24.3

Table S3: TGA data of PF4SN donor-accepter copolymers. Glass transition temperature (T_g), onset temperature of degradation (T_{onset}), peak temperature (T_{peak}), the experimentally determined weight loss (Wt) of the first degradation event, and the calculated theoretical weight loss (Theory) associated with complete removal of the neopentyl protecting groups. A T_g was not detected (n.d.) for PF4SN-T.

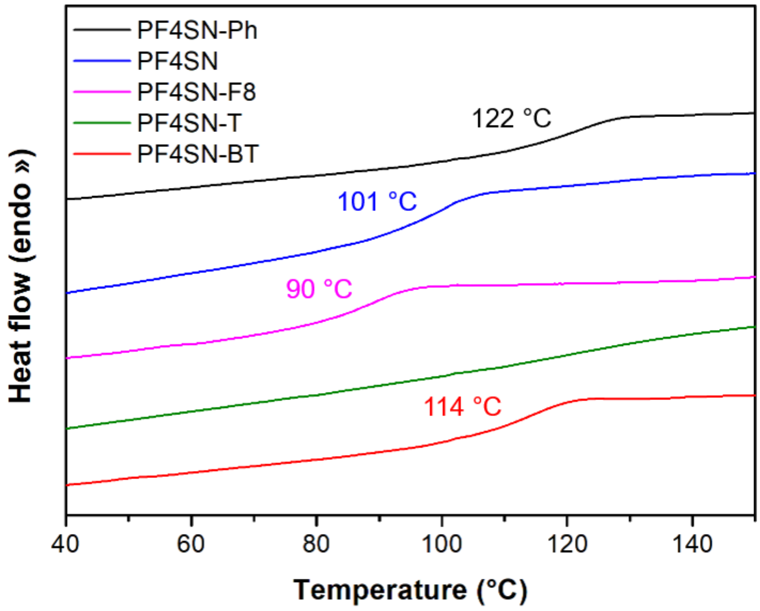

Figure S30: DSC thermograms of the PF4SN copolymers. Temperatures indicate the glass transition temperature (T_g); a T_g could not be identified for P4SN-T.

3.6 Structural properties

Room temperature WAXS measurements were performed to investigate the structural properties of the PF4SN copolymers. First, thermally annealed powder of the simplest polymer PF4SN was compared to in-house synthesized PF8 (**Figure S31a**). The broad scattering patterns show great similarities to both the nematic and noncrystalline β-phase typically observed in PF8.[17,18] Although the applied q-range is too narrow to distinguish the two, a nematic glass is more likely, since the β-phase is usually only observed in solvent-treated samples and is unique for PF8.[19] Diffraction of such frozen liquid crystals is the result of stacking of ribbonlike macromolecules, where the peak maximum observed around 13.7 nm^{-1} (0.46 nm) corresponds to the thickness of the ribbons and the weak shoulder at lower q-values to stacking of the side chains in the direction of the polymer backbone.[17] The width of the ribbons falls outside of the selected q-range. Interestingly, annealing of the as-obtained powders well above the T_g hardly had any effect; only a slightly increased intensity due to a somewhat improved ordering was recognized (**Figure S32**).

The same behavior was found for the other PF4SN copolymers: only broad and featureless diffraction patterns were identified (**Figure S33**). Compared to the alkylated copolymers, all PF4SN copolymers demonstrated broader and weaker signals, implying worse ordering (**Figure S31**). Furthermore, peak maxima were always centered around lower q-values (i.e., larger Bragg spacings), presumably being a result of the bulky side groups giving rise to ribbons with larger dimensions. Overall, the absence of any crystalline phases in the PF4SN copolymers confirms the DSC data, as no additional transitions were identified besides the T_g. Because in addition to the neopentyl protected sulfonate groups, the molecular weight, end groups, and processing conditions can inhibit crystallization as well, further investigation is still required to obtain a better understanding of the structural behavior.

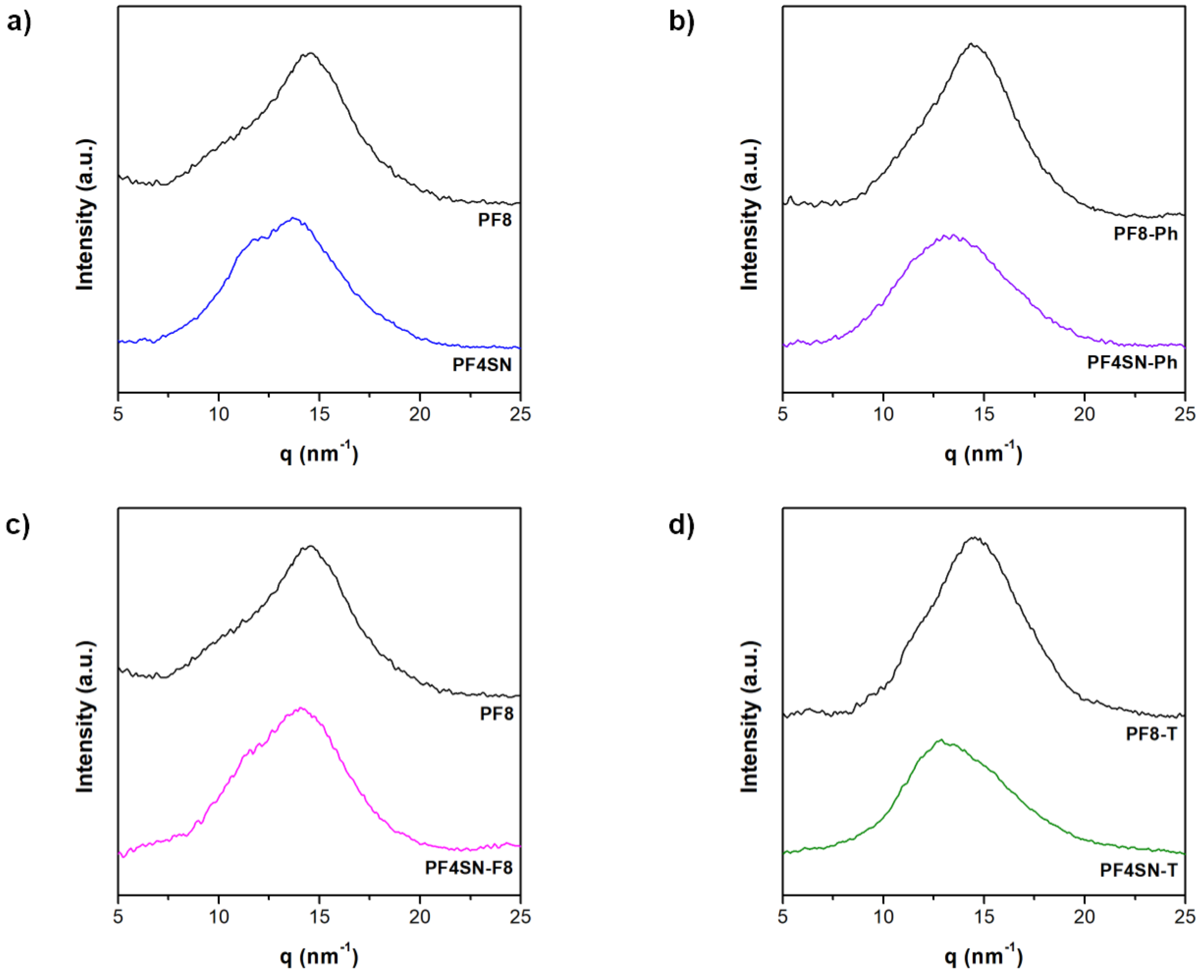

Figure S31: Stacked WAXS patterns of neopentyl-protected PF4SN copolymers and their alkylated F8 analogues. (a) PF4SN and PF8, (b) PF4SN-Ph and PF8-Ph, (c) PF4SN-F8 and PF8, and (d) PF4SN-T and PF8-T. All copolymers were annealed at 130 °C for 1 h prior to the measurement.

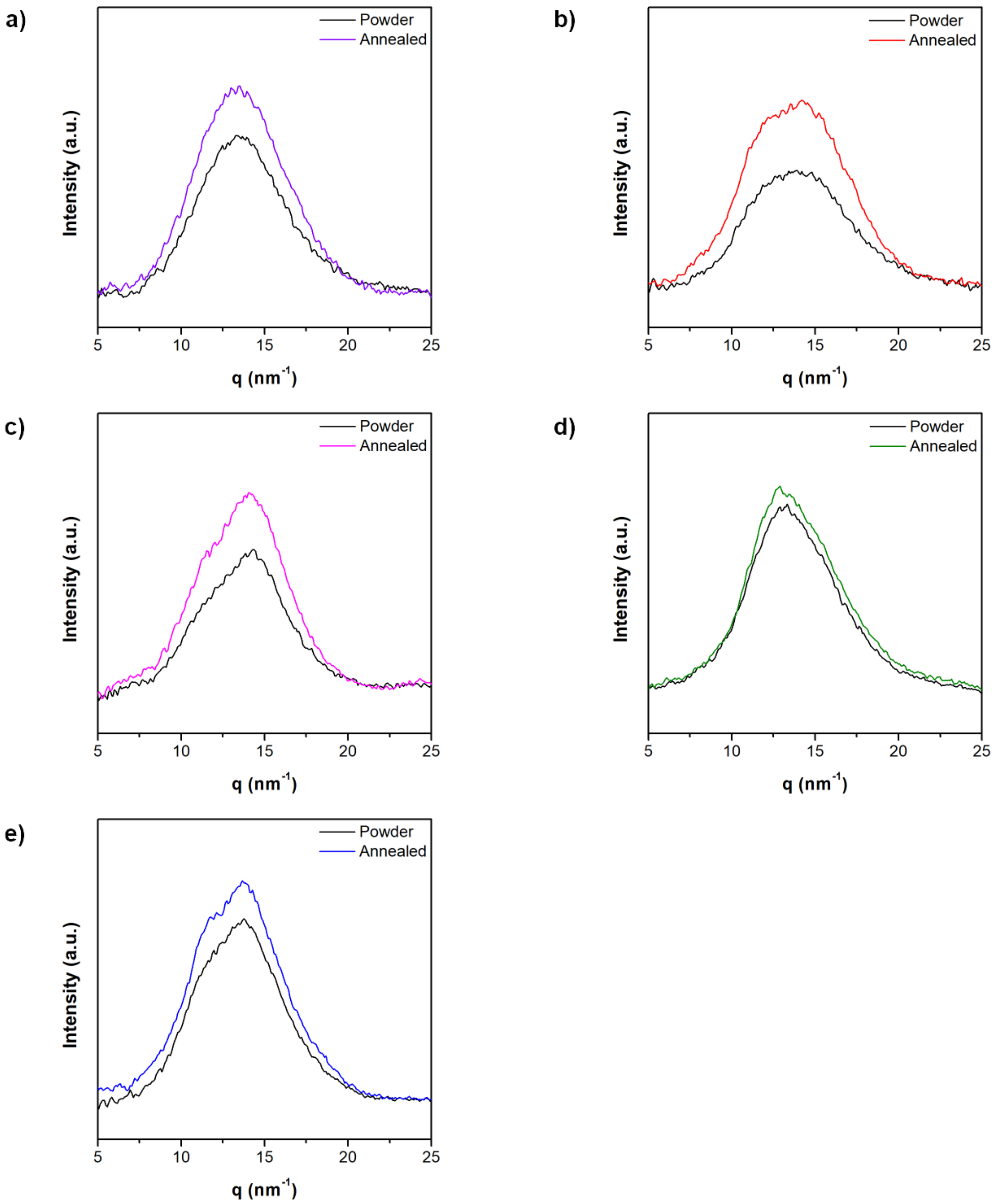

Figure S32: Comparison of the WAXS profiles of the as-obtained and annealed (130 °C for 1 h) PF4SN copolymer powders. (a) PF4SN-Ph, (b) PF4SN-BT, (c) PF4SN-F8, (d) PF4SN-T, and (e) PF4SN.

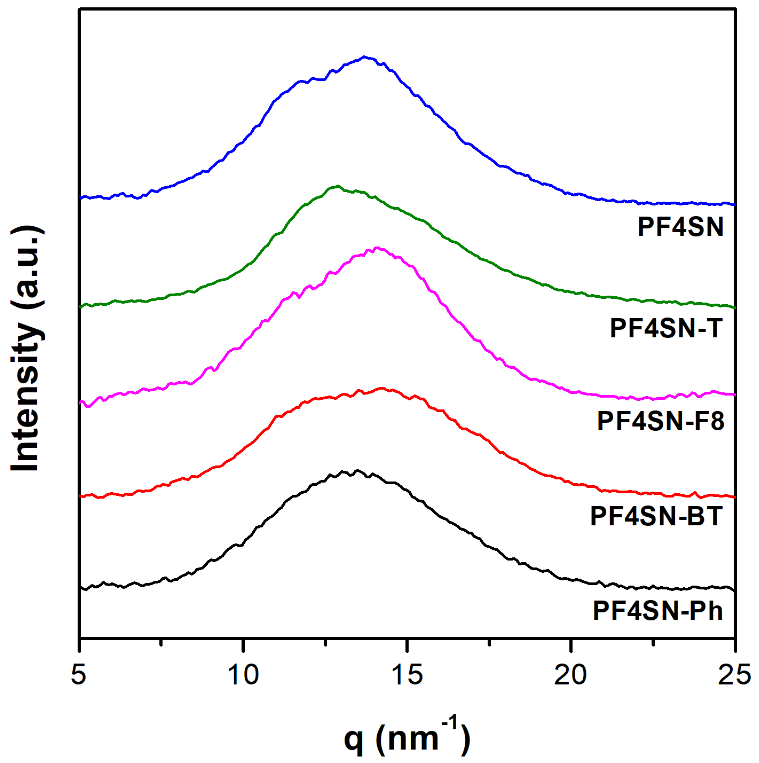

Figure S33: Stacked WAXS profiles of all PF4SN copolymers. The copolymers were annealed at 130 °C for 1 h prior to the measurement.

4 Literature comparison

Entry	CPE	Protecting group	Deprotection route	Conversion (%)	UV-Vis	PL	Reference
1	PP	Neopentyl	$(C_2H_5)_2NH.HBr$ (120 °C, 48 h)	ND	N (-)	N (-)	23
2	PPE	TFMB	NaOH (101 °C, 24 h)	ND	Y ?	Y ?	24
3	PT	Neopentyl	Thermal (185 °C, 10 min)	ND	Y ✗	N (-)	25
4	PT	Isobutyl	NaI (80 °C, 6 h)	ND	Y ✗	N (-)	26
5	PT	Neopentyl	LiBr (80 °C, 8 days)	ND	N (-)	N (-)	27
6	PF	Neopentyl	NaN_3 (130 °C, 22 h)	99+	Y ✓	Y ✓	This work

Table S4: Comparison of this work (entry 6) to recent literature where protection chemistry was used for the preparation of sulfonate-functionalized CPEs. Applied protecting groups, the route, and conversion (i.e., the degree of deprotection) are reported in this overview. UV-Vis and PL columns summarize whether UV-Vis and PL spectroscopy were performed (Yes or No) and, when applicable, if the photophysical properties remain unchanged after deprotection (✓ or ✗ or ?). References refer to the numbers listed in the main text. Used abbreviations: PP (polyphenylene), PPE (poly(phenylene ethynylene)), PT (polythiophene), PF (polyfluorene), TFMB (α-trifluoromethylbenzyl), and ND (not determined).

5 PF8 copolymer synthesis

Entry	Polymer	Method	Yield (%)	M_n	X_n	Đ
1	PF8	C	61	5.94	15.3	4.34
2	PF8-Ph	A	81	5.49	11.8	2.81
3	PF8-T	A	76	2.85	6.1	1.82
4	PF8-BT	A	67	3.60	6.9	2.27

Table S5: Characteristics of the alkylated PF8 copolymers used for comparative studies. Details about the synthesis route (A or C) are described in the "Polymerizations" section. Yields were determined gravimetrically after precipitation and drying of the product, and molecular weights M_n (kg mol^{-1}) and their distribution Đ were determined by GPC (PS standards). X_n is the number-average degree of polymerization, calculated from M_n using the mass of the repeating unit.

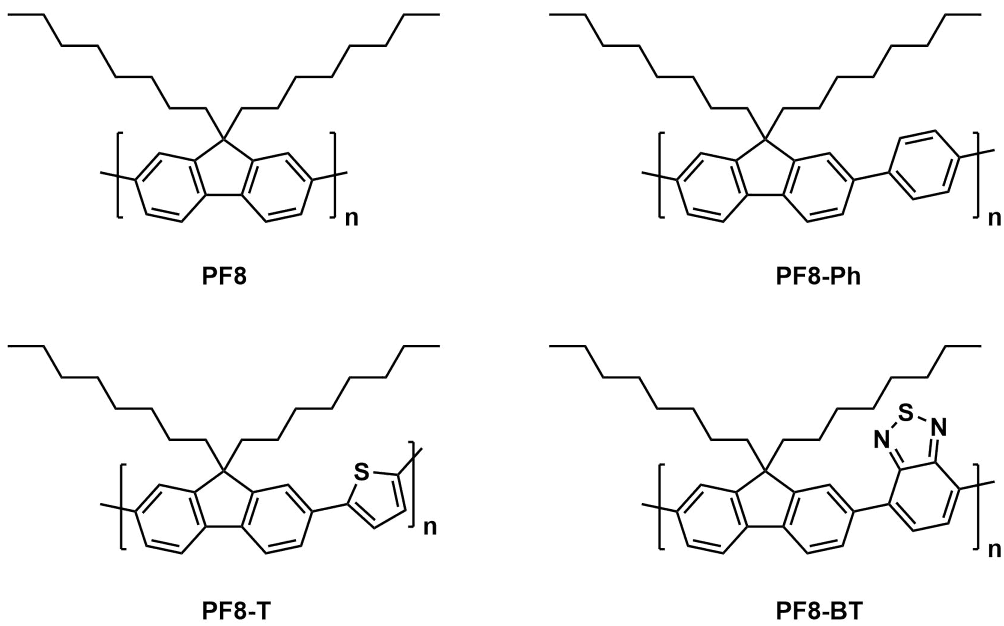

Figure S34: Chemical structures of the hydrophobic PF8 copolymers used for comparative studies (PF8, PF8-Ph, PF8-T, and PF8-BT).

References

1. Mai, C.; Zhou, H.; Zhang, Y.; Henson, Z. B.; Nguyen, T.; Heeger, A. J.; Bazan, G. C. Facile Doping of Anionic Narrow-Band-Gap Conjugated Polyelectrolytes During Dialysis. *Angew. Chem. Int. Ed.* **2013**, *52*, 12874–12878.

2. Yang, Q.; Sheng, M.; Henkelis, J. J.; Tu, S.; Wiensch, E.; Zhang, H.; Zhang, Y.; Tucker, C.; Ejeh, D. E. Explosion Hazards of Sodium Hydride in Dimethyl Sulfoxide, *N,N*-Dimethylformamide, and *N,N*-Dimethylacetamide. *Org. Process Res. Dev.* **2019**, *23*, 2210–2217.

3. Hofman, A. H.; Fokkink, R.; Kamperman, M. A Mild and Quantitative Route Towards Well-Defined Strong Anionic/Hydrophobic Diblock Copolymers: Synthesis and Aqueous Self-Assembly. *Polym. Chem.* **2019**, *10*, 6109–6115.

4. Hofman, A. H.; Pedone, M.; Kamperman, M. Protected Poly(3-Sulfopropyl Methacrylate) Copolymers: Synthesis, Stability, and Orthogonal Deprotection. *ACS Polym. Au* **2022**, *2*, 169–180.

5. Shimizu, S.; Imamura, Y.; Ueki, T. Incompatibilities between *N*-Bromosuccinimide and Solvents. *Org. Process Res. Dev.* **2014**, *18*, 354–358.

6. Ishiyama, T.; Murata, M.; Miyaura, N. Palladium(0)-Catalyzed Cross-Coupling Reaction of Alkoxydiboron with Haloarenes: A Direct Procedure for Arylboronic Esters. *J. Org. Chem.* **1995**, *60*, 7508–7510.

7. Aloi, A.; Guibert, C.; Olijve, L. L. C.; Voets, I. K. Morphological Evolution of Complex Coacervate Core Micelles Revealed by iPAINT Microscopy. *Polymer* **2016**, *107*, 450–455.

8. Nielsen, K. T.; Bechgaard, K.; Krebs, F. C. Removal of Palladium Nanoparticles from Polymer Materials. *Macromolecules* **2005**, *38*, 658–659.

9. Gallagher, W. P.; Vo, A. Dithiocarbamates: Reagents for the Removal of Transition Metals from Organic Reaction Media. *Org. Process Res. Dev.* **2015**, *19*, 1369–1373.

10. Izumi, A.; Nomura, R.; Masuda, T. A New Synthetic Method for Poly(arylene)s Using Bis(pinacolato)diboron as a Condensation Reagent. *Chem. Lett.* **2000**, *29*, 728–729.

11. Walczak, R. M.; Brookins, R. N.; Savage, A. M.; van der Aa, E. M.; Reynolds, J. R. Convenient Synthesis of Functional Polyfluorenes via a Modified One-Pot Suzuki-Miyaura Condensation Reaction. *Macromolecules* **2009**, *42*, 1445–1447.

12. Brouwer, F.; Alma, J.; Valkenier, H.; Voortman, T. P.; Hillebrand, J.; Chiechi, R. C.; Hummelen, J. C. Using Bis(pinacolato)diboron to Improve the Quality of Regioregular Conjugated Co-polymers. *J. Mater. Chem.* **2011**, *21*, 1582–1592.

13. Kolomanska, J.; Johnston, P.; Gregori, A.; Domínguez, I. F.; Egelhaaf, H.; Perrier, S.; Rivaton, A.; Dagron-Lartigau, C.; Topham, P. D. Design, Synthesis and Thermal Behaviour of a Series of Well-Defined Clickable and Triggerable Sulfonate Polymers. *RSC Adv.* **2015**, *5*, 66554–66562.

14. Müller, C. On the Glass Transition of Polymer Semiconductors and Its Impact on Polymer Solar Cell Stability. *Chem. Mater.* **2015**, *27*, 2740–2754.

15. Xie, R.; Weisen, A. R.; Lee, Y.; Aplan, M. A.; Fenton, A. M.; Masucci, A. E.; Kempe, F.; Sommer, M.; Pester, C. W.; Colby, R. H.; Gomez, E. D. Glass Transition Temperature from the Chemical Structure of Conjugated Polymers. *Nat. Commun.* **2020**, *11*, 893.

16. Grova, I. R.; Macedo, A. G.; Roman, L. S.; Akcelrud, L. Correlations Between the Number of Thiophene Units and the Photovoltaic Behavior of Fluorene-Oligothiophene Copolymers. *Eur. Polym. J.* **2013**, *49*, 3539–3547.

17. Grell, M.; Bradley, D. D. C.; Ungar, G.; Hill, J.; Whitehead, K. S. Interplay of Physical Structure and Photophysics for a Liquid Crystalline Polyfluorene. *Macromolecules* **1999**, *32*, 5810–5817.

18. Chen, S. H.; Su, A. C.; Chen, S. A. Noncrystalline Phases in Poly(9,9-di-*n*-octyl-2,7-fluorene). *J. Phys. Chem. B* **2005**, *109*, 10067–10072.

19. Knaapila, M.; Winokur, M. J. Structure and Morphology of Polyfluorenes in Solutions and the Solid State. *Adv. Polym. Sci.* **2008**, *212*, 227–272.
